# Huanglian-Wendan Decoction alleviates DSS-induced colitis by modulating the gut microbiota and protecting against intestinal injury via suppression of colonic apoptosis and endoplasmic reticulum stress

**DOI:** 10.1007/s13659-026-00636-w

**Published:** 2026-07-06

**Authors:** Liang Li, Xinyi Zhan, Hidayat Ullah, Wenxian Guo, Ping Gui, Weijie Peng, Quanxi Mei, Weibo Dai, Yuting Duan, Xia Yuan, Xianjing Hu

**Affiliations:** 1 Pharmacology Laboratory, Zhongshan Hospital, Guangzhou University of Chinese Medicine, Zhongshan, 528401 Guangdong PR China; 2 Dongguan Key Laboratory of TCM for Prevention and Treatment of Refractory Digestive Diseases, Guangdong Provincial Key Laboratory of Natural Drugs Research and Development, Guangdong Medical University, Dongguan, 523808 PR China; 3 Dongguan Key Laboratory of Fundamental Research and Clinical Application of Toxic Chinese Medicine, The First Dongguan Affiliated Hospital, School of Pharmacy, Guangdong Medical University, Dongguan, 523121 PR China; 4 School of Pharmacy, Dongguan Branch, National Engineering Research Center for Modernization of Traditional Chinese Medicine, Guangdong Medical University, Dongguan, 523808 PR China; 5 Department of Proctology, The First Dongguan Affiliated Hospital, Guangdong Medical University, Dongguan, 523121 PR China; 6 Evidence-Based Medicine Center, The Affiliated Traditional Chinese Medicine Hospital, Guangzhou Medical University, Guangzhou, 510140 PR China; 7 Cancer Center, The First Huizhou Affiliated Hospital of Guangdong Medical University, Huizhou, 516000 PR China

**Keywords:** Inflammatory bowel disease, Chinese medicine formula, Huanglian-Wendan Decoction, Apoptosis, Endoplasmic stress, Gut microbiota

## Abstract

Inflammatory bowel disease (IBD) is a chronic disorder characterized by recurrent intestinal inflammation and gut microbiota dysbiosis. Huanglian-Wendan Decoction (HLWDD) has been clinically used for IBD treatment; however, its underlying mechanisms remain unclear. In this study, a dextran sulfate sodium (DSS, 2.25%)-induced IBD mouse model was established to evaluate the therapeutic effects of HLWDD. The protective mechanisms were investigated in colon tissues of DSS-induced mice using ELISA, immunoblotting, histological, and immunohistochemical analyses. In addition, the impact of HLWDD on gut microbiota dysbiosis was analyzed using 16S rRNA sequencing. Antibiotic treatment was applied before DSS administration to deplete gut microbiota and verify the role of microbial modulation. Furthermore, the phytochemical constituents of HLWDD were characterized using liquid chromatography–quadrupole time-of-flight tandem mass spectrometry (LC-QTOF-MS/MS). The results demonstrated that HLWDD markedly alleviated DSS-induced colitis, as evidenced by reduced body weight loss, rectal bleeding, colon shortening, and disease activity index (DAI) scores. Mechanistically, HLWDD suppressed inflammatory responses in colon tissues by inhibiting the TLR4/MyD88/NF-κB and IL-6/JAK2/STAT3 signaling pathways, while enhancing epithelial barrier integrity through upregulation of ZO-1, Occludin, Claudin-1, and Mucin-2. In addition, HLWDD attenuated endoplasmic reticulum stress (ERS) and apoptosis by downregulating CHOP, phospho-eIF2α, cleaved caspase-3, and Bax, while increasing Bcl-2 expression in colonic tissues. Microbiota analysis revealed an increased abundance of beneficial bacterial genera such as *Akkermansia* and *Escherichia-Shigella*–related commensals, along with enrichment of beneficial bacterial families including *Ruminococcaceae*, *Lachnospiraceae*, and *Verrucomicrobiaceae*, whereas potentially harmful taxa such as *Escherichia* and *Paraprevotella* were reduced. HLWDD also increased the production of short-chain fatty acids (SCFAs), including acetate, butyrate, and isobutyrate, thereby promoting intestinal homeostasis. Importantly, the protective effects of HLWDD were largely abolished following antibiotic-mediated gut microbiota depletion, confirming the essential role of microbial modulation in its therapeutic action. Collectively, these findings suggest that HLWDD ameliorates IBD by regulating gut microbiota composition and function, thereby inhibiting colonic ER stress and apoptosis and restoring intestinal barrier integrity. This study provides mechanistic evidence supporting the potential clinical application of HLWDD as a novel therapeutic strategy for IBD.

**Graphical Abstract:**

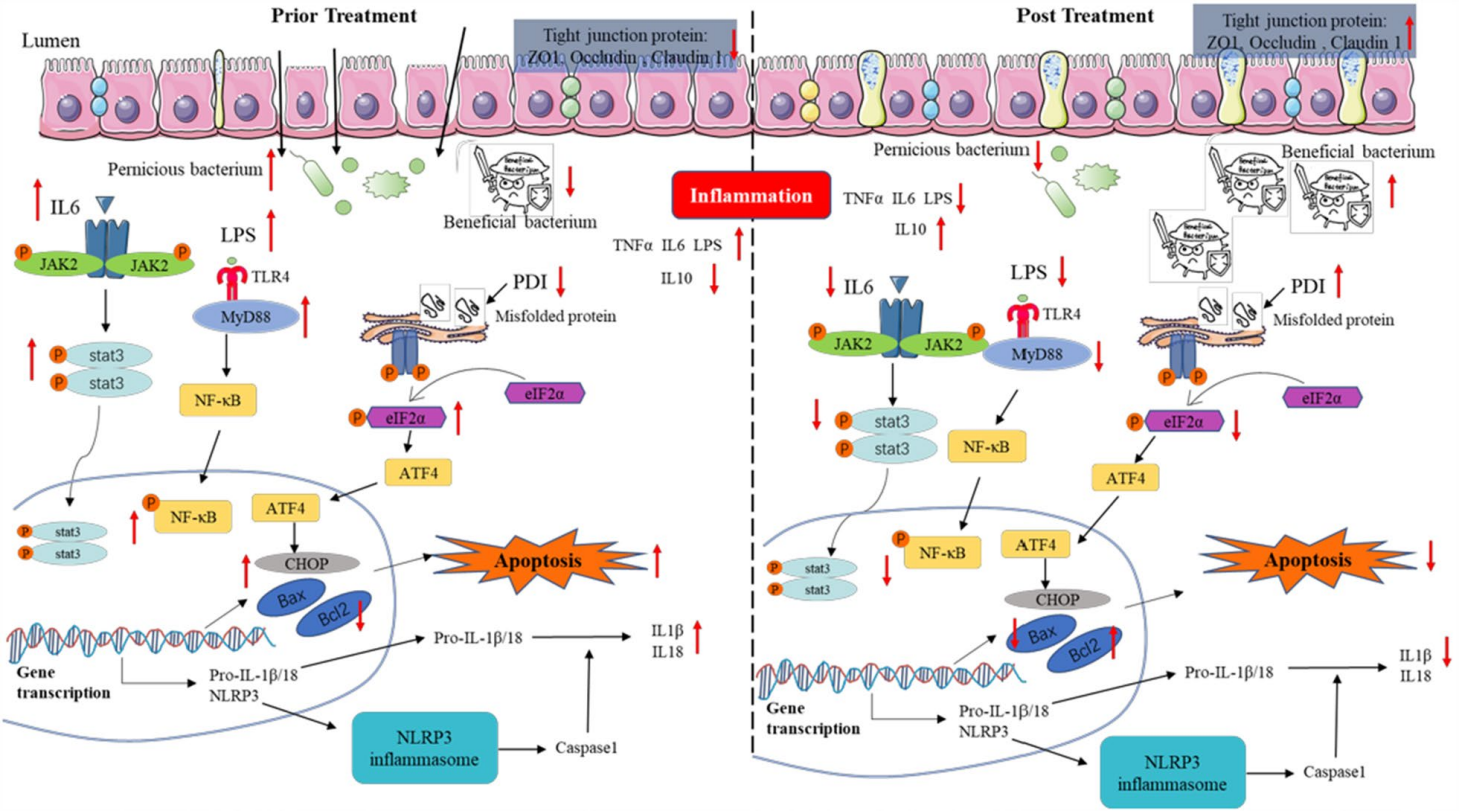

**Supplementary Information:**

The online version contains supplementary material available at 10.1007/s13659-026-00636-w.

## Introduction

Inflammatory bowel disease (IBD) is a global disorder that profoundly affects both physical and mental health, with epidemiological data suggesting that since 1996, roughly 1 in 200–300 individuals worldwide has been diagnosed with the condition [1, 2], and the incidence is still increasing rapidly. The number of IBD patients in China is projected to exceed 1.5 million by 2025 [3]. IBD has been reported to markedly increase the risk of developing complications such as arthritis, osteoporosis, secondary liver injury, and even colon cancer [4–6]. Current therapeutic interventions for inflammatory bowel disease (IBD) mainly include 5-aminosalicylic acids (5-ASA), immunomodulatory agents, corticosteroids, and biologic therapies [7]. However, these treatments primarily focus on symptom management rather than disease resolution, and long-term use of such medications may increase the risk of lymphoma. Therefore, there is a growing need to develop safer and more effective therapeutic strategies for the management of IBD [8]. There is a growing demand for new pharmacological agents and traditional or ethnomedicinal approaches.

The precise pathogenesis of IBD remains elusive, but endoplasmic reticulum stress (ERS) has recently emerged as an important contributor to intestinal inflammation and epithelial dysfunction. ERS occurs when the protein-folding capacity of the endoplasmic reticulum is disrupted, leading to activation of the unfolded protein response (UPR) and inflammatory signaling pathways [9]. In intestinal epithelial cells, ERS can downregulate the MUC2 expression, impairing the mucosal barrier and perpetuating a cycle of chronic intestinal inflammation [10]. Persistent ERS has been shown to promote epithelial cell apoptosis, disrupt intestinal barrier integrity, and exacerbate inflammatory responses, thereby contributing to the progression of IBD [11].

IBD is characterized by a chronic inflammatory environment that is closely associated with the gut microbial and immune milieu. Chronic inflammation perturbs the gut microbiota, leading to a reduction in beneficial bacteria and an expansion of pathogenic species [12]. Research has shown that *Fusobacterium nucleatum* can exacerbate inflammation in normal epithelial cells and promote epithelial-to-mesenchymal transition (EMT) [13]. *Salmonella enterica* was involved in the inflammation of the intestine, which persisted after pathogen clearance and irreversibly escalated in severity with repeated infections [14]. Studies have suggested that fecal microbiota transplantation (FMT) and probiotics may offer potential therapeutic approaches for IBD treatment [15, 16]. Therefore, regulating intestinal microbiota can be a key strategy for treating IBD. Recent studies further indicate that gut microbiota dysbiosis is closely linked to ERS activation in intestinal epithelial cells. Microbial metabolites, such as short-chain fatty acids (SCFAs), can regulate ER homeostasis and intestinal barrier function, whereas pathogenic bacteria may aggravate ER stress and inflammatory signaling pathways [17]. Short-chain fatty acids produced by beneficial gut bacteria have been reported to maintain intestinal epithelial integrity and regulate inflammatory responses, thereby contributing to intestinal homeostasis [18]. Therefore, the interaction between gut microbiota dysbiosis and ER stress is increasingly recognized as an important mechanism contributing to intestinal inflammation and epithelial damage in IBD, and targeting these pathways may represent a promising therapeutic strategy. Traditional herbal formulations, such as Huanglian-Wendan Decoction, are known to contain multiple bioactive compounds that can simultaneously modulate inflammatory signaling, alleviate endoplasmic reticulum stress, and restore gut microbial balance. This multi-target capability provides a strong rationale for investigating HLWDD as a potential therapeutic strategy in IBD.

Huanglian-Wendan Decoction (HLWDD), a classical formulation of traditional Chinese medicine, was first recorded in the Tang Dynasty text Beiji Qianjin Yaofang by Sun Simiao [19]. It has been commonly used to treat insomnia and gastritis for thousands of years [20]. Recently, it has been found to treat hypertension, coronary heart disease, diabetes, and liver cancer [21, 22]. Our recent clinical study showed that HLWDD could alleviate IBD in patients in the clinic [23], although the detailed mechanism remains unclear.

Despite growing evidence that ER stress and gut microbiota dysbiosis contribute to IBD pathogenesis, it remains unclear whether multi-component herbal formulations such as Huanglian-Wendan Decoction (HLWDD) can simultaneously target these pathways to confer therapeutic benefits. Therefore, in this study, we aimed to investigate whether HLWDD ameliorates DSS-induced colitis by modulating colonic ER stress, epithelial apoptosis, and gut microbiota composition, and to determine the essential role of gut microbiota in mediating its protective effects.

## Materials and methods

### Chemicals and reagents

Dextran sulfate sodium (DSS, MW: 36 ⁓ 50 kDa, S5036, 9011-18-1) was obtained from MP Biomedicals Inc. (California, USA). Salicylazosulfapyridine (SASP, 33816) was purchased from MedChemExpress (New Jersey, USA). Elisa kits, including DAO (252210519), MPO (411210519), IL-1β (370210610), IL-10 (371210513), TNFα (569211010), LPS (261211222), IL-6 (385210923), were secured from Tianjin Anoric Biotechnology CO., Ltd (Tianjin, China), and NAG (202111) was purchased from MSKBIO (Wuhan, China). Primary antibodies against Claudin 1 (AF0127), Occludin (DF7504), ZO1 (AF5145), MyD88 (AF5195), JAK2 (AF6022), STAT3 (AF6294), cleaved PARP (AF7023), Bax (AF0120), Bcl2 (AF6139), and β-actin (AF7018) were purchased from Affinity Biosciences Ltd (OH, USA). Primary antibodies against p-NF-κB (3033T), NF-κB (8242s), p-JAK2 (D15E2), cleaved caspase 3 (9661), PDI (3501), CHOP (2895), and p-eIF2α (9721S) were obtained from Cell Signal Technology Inc. (Boston, USA). Primary antibodies against TLR4 (293072) and p-STAT3 (sc-8059) were supplied by Santa Cruz Biotechnology Inc. (California, USA), and eIF2α (11233-I-AP) was obtained from Proteintech Group Inc. (Chicago, USA), respectively. All antibodies were diluted in 1:1000, while p-STAT3 was diluted in 1:500.

### Preparation of HLWDD

HLWDD was composed of Coptis chinensis Franch. (10 g), Pinellia ternata(Thunb) Breit. (10 g), Bambusa tuldoides Munro. (10 g), Citrus sinensis Osbeck. (10 g), Citrus reticulata Blanco. (10 g), Poria cocos (Schw.) Wolf. (10 g), Glycyrrhiza uralensis Fisch. (5 g). Coptis chinensis Franch. (20200301, Sichuan), Citrus sinensis Osbeck (20200501, Jiangxi) and Glycyrrhiza uralensis Fisch. (20190709, Gansu) were purchased from Sinopharm Group Feng Liaoxing Medicinal Herbs Decoction and Pieces Co., LTD (Foshan, China). Pinellia ternata(Thunb) Breit. (200206, Sichuan) and Bambusa tuldoides Munro (191102, Guangdong) were purchased from Yulin Bencaotang TCM Decoction and Pieces Co. Ltd. (Yulin, China). Citrus reticulata Blanco (200301) and Poria cocos (Schw.) Wolf (20052501) was purchased from Guangzhou Zhixin Chinese Medicine Decoction and Pieces Co., LTD (Guangzhou, China) and Zhongshan Tongxin Pharmaceutical Co., LTD. Chinese medicine decoction and pieces of a branch (Yunnan, China), respectively. A total of 2925 g of HLWDD raw materials were soaked in distilled water for 1 h, boiled with 10 folds of water (v/w) for 1 h, and filtered to get the filtrate, and the residue was boiled once more in 8 folds of water (v/w) for 1 h. The filtrate was combined and concentrated to the indicated concentration. According to the clinical dose, each patient took 65 g of Huanglian Wendan Decoction every day (estimated from the mass of crude material), meaning that the dosage was 0.929 g/kg for humans, which was converted to a mouse dosage of 8.45 g/kg according to the dose conversion factor.

### Experimental design

**Fig. 1.**
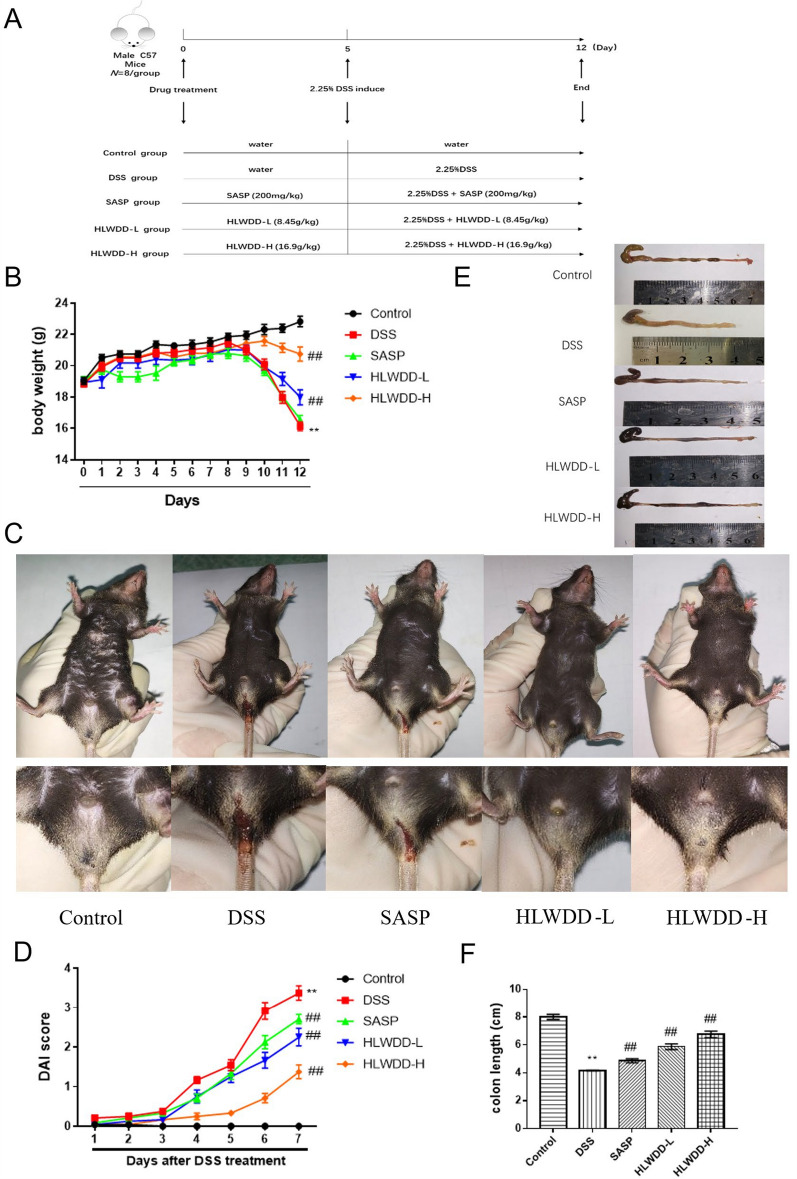
Huanglian-Wendan Decoction (HLWDD) alleviated DSS-induced IBD in mice. **A** Design for HLWDD in anti-IBD effect on the mouse model induced by DSS. **B** Effect of HLWDD (8.45, 16.9 g/kg) and SASP (200 mg/kg) on the body weight of mice. **C** Representative photographs of the anal region of mice . **D** Disease activity index of IBD mice. **E** Representative photographs of the colon. **F** Effect of HLWDD and SASP on the colon length of IBD mice. Data are represented as mean ± SEM, and statistical significance was analyzed by one-way ANOVA, ^*^p < 0.05, ^**^p < 0.01, vs. the control group; ^#^*p* < 0.05, ^##^*p* < 0.01, vs. the model group (DSS only)

The animal experiments were reviewed and approved by the Institutional Animal Care and Use Committee of Zhongshan Hospital of TCM (AEWC-2021041) following the standards set by the principles of Laboratory Animal Care and the guidelines of the Zhongshan Hospital of TCM Animal Research Committee. Eight-week-old male C57BL/6 mice were obtained from Guangdong Laboratory Animal Center (Foshan, China), housed under a 12 h light/dark cycle, and provided with food and water ad libitum. Mice were randomly assigned to five groups (n = 8 per group) using a computer-generated random number table: control, model (2.25% DSS), SASP (200 mg/kg), HLWDD low-dose (HLWDD-L, 8.45 g/kg, based on crude material), and HLWDD high-dose (HLWDD-H, 16.9 g/kg). The selection of HLWDD doses was based on clinical equivalent dose conversion. According to guidelines for dose translation between humans and animals based on body surface area normalization recommended by the FDA, the human-to-rat equivalent dose ratio is 6.25:1. Given that the clinical dosage of HLWDD in adults is 2.271 g·kg⁻^1^·day⁻^1^ [24]. The calculated rat equivalent dose is 14.19 g·kg⁻^1^·day⁻^1^ (approximately 14.2 g/kg). Based on this equivalent dose, two experimental doses were selected to evaluate the pharmacological effects of HLWDD. The high dose was set at 16.9 g/kg, which is slightly higher than the calculated equivalent dose to ensure therapeutic efficacy, while the low dose was set at 8.45 g/kg, corresponding to 50% of the high dose, to evaluate dose-dependent effects. To induce acute colitis, all groups except the control were administered 2.25% w/v DSS (molecular weight 36–50 kDa) in autoclaved drinking water from day 5 to day 12. SASP and HLWDD were administered orally from day 0 to day 12. The study was performed in two independent batches: a pilot study for DSS model optimization and HLWDD dosage determination, followed by the main experiment to evaluate therapeutic effects and underlying mechanisms. In both batches, the following indicators were measured: clinical parameters (body weight, DAI, colon length), histology and immunohistochemistry of intestinal barrier proteins (ZO-1, Occludin, Claudin-1, Mucin-2), cytokine levels and protein expression (Western blot for inflammatory, ER stress, and apoptosis markers), and gut microbiota analysis (16S rRNA sequencing of fecal samples). Following the treatment period, mice were euthanized by anesthetic overdose using 1% Nembutal (sodium pentobarbital, 50 mg/kg, intraperitoneally) to ensure a humane endpoint, followed by cervical dislocation as a secondary confirmation of death. The overall experimental design is summarized in Fig. 1A.

### Antibiotic treatment

Antibiotic treatment was carried out following the modified method described previously [15]. In brief, eight-week-old male C57BL/6 mice were randomly assigned using a computer-generated random number table to the following groups: control, 2.25% DSS (DSS), 2.25% DSS + HLWDD (16.9 g/kg), Control + antibiotics (Control_ABX), 2.25% DSS + antibiotics (DSS_ABX), and 2.25% DSS + HLWDD + antibiotics (HLWDD_ABX). To deplete the intestinal microbiota, mice in the antibiotic-treated groups received a broad-spectrum antibiotic cocktail consisting of vancomycin (0.5 g/L, Aladdin, China), metronidazole (1 g/L, Aladdin, China), neomycin sulfate (1 g/L, Aladdin, China), and ampicillin (1 g/L, Solarbio, China) dissolved in drinking water and provided ad libitum from day 1 to day 5. HLWDD was administered orally from day 6 to day 17. The colitis mouse model was induced by administering 2.25% DSS (w/v) in drinking water for 7 days (from day 10 to day 17). At the end of the treatment period, mice were euthanized with an overdose of 1% Nembutal (sodium pentobarbital, 50 mg/kg, intraperitoneally) followed by cervical dislocation to ensure death.

### Assessment of disease activity index (DAI)

The Disease Activity Index (DAI) was used to evaluate the severity of colitis by monitoring changes in body weight, stool consistency, and the presence of blood in the stool, according to a previously described protocol [25]. Each of these three indicators was scored individually according to Table 1. The final DAI for each mouse was calculated as the average of the three scores (range 0–4), allowing partial scoring when only one or two criteria were affected. Higher DAI scores indicate more severe disease activity. The criteria for DAI scoring are summarized in Table 1.

**Table 1:** Scoring system for DAI

Score	Weight loss	Stool consistency	Blood stool
0	None	Normal	None
1	1~5%	Soft stool	Slight occult blood
2	5~10%	Paste stool	Occult blood
3	10~15%	Loose stool	Bleeding
4	>15%	Diarrhea	Gross bleeding

### Sample collection

After treatment, stool samples were collected for 16S rRNA sequencing. The mice were then anesthetized, and blood was drawn from serum following centrifugation at 10000 rpm for 10 min at 4°C. The mice were sacrificed, the distal colon segment (approximately 1–2 cm from the anus) was swiftly removed, and its length was measured. A portion of this distal colon was fixed in 4% paraformaldehyde (PFA) for histological staining, while the remaining tissue was immediately snap-frozen and stored at −  80 °C for subsequent molecular analyses.

### Enzyme-linked immunosorbent assay (ELISA)

ELISA kits were procured from commercial vendors, and biochemical assessments were executed in accordance with the protocols delineated by the manufacturers. In summary, colon tissues were rinsed utilizing cold phosphate-buffered saline (PBS, 0.01M, pH 7.4), subsequently homogenized with a tenfold volume of PBS (incorporating 1% protease inhibitor, v/w) at a temperature of 4℃, and were then subjected to centrifugation at 10,000 rpm for a duration of 10 minutes. The resultant supernatants were isolated for biochemical analysis.

### Western blot analysis

Colon tissue samples were rinsed with ice-cold PBS and lysed in radioimmunoprecipitation assay (RIPA) buffer supplemented with protease and phosphatase inhibitors (Beyotime, China) for 30 minutes on ice. Following centrifugation at 15,000 rpm for 10 minutes at 4℃, the samples were harvested, and a BCA protein assay kit was employed to quantify protein concentrations. An equivalent quantity of protein (40 μg) was applied and resolved on 8–15% sodium dodecyl sulfate-polyacrylamide gels, followed by transfer onto polyvinylidene difluoride (PVDF) membranes (Merck Millipore Ltd. IPVH00010, R1PB86944, Darmstadt, Germany). The membranes underwent a blocking procedure utilizing QuickBlockTM solution (P0252, Beyotime, Shanghai, China) at ambient temperature for 15 minutes, followed by washing in PBST buffer, and were incubated overnight at 4℃ with primary antibodies targeting Claudin1, Occludin, ZO1, TLR4, MyD88, NF-κB, phosphor-NF-κB, JAK2, phosphor-JAK2, STAT3, phosphor-STAT3, cleaved PARP, cleaved caspase3, Bcl2, Bax, PDI, CHOP, phosphor-eIF2a, eIF2a, and β-actin, diluted in TBST containing 5% bovine serum albumin (BSA). Following three washes with PBST, the membranes were incubated with the appropriate secondary antibodies conjugated to horseradish peroxidase (HRP) (1:10,000) at room temperature for 1.5 hours. Protein bands were visualized using an enhanced chemiluminescence (ECL) kit, and Quantitative analyses were performed using ImageJ.

### 16S rRNA gene high-throughput sequencing

Stool samples were collected from the colon into 1.5 mL sterile EP tubes and immediately stored at −  80 °C before being sent to BGI.tech (http://www.bgitechsolutions.com/) for 16S rRNA gene sequencing. Genomic DNA was extracted from colon contents, and 30 ng of DNA was used for PCR amplification. The V3–V4 hypervariable region of the 16S rRNA gene was amplified using the amplified by PCR using specific primers (5′-ACTCCTACGGGAGGCAGCA-3′ and 5′-GGACTACHVGGGTWTCTAAT-3′) for 25 cycles. PCR amplicons were purified with Agencourt AMPure XP magnetic beads and resuspended in Elution Buffer for library construction [26]. The library fragment size and concentration were assessed using an Agilent 2100 Bioanalyzer, and qualified libraries were sequenced on the Illumina HiSeq platform. Raw reads were merged into Tags based on overlapping sequences, clustered into operational taxonomic units (OTUs) at 97% similarity, and annotated using the SILVA 138 database. Bioinformatics analysis was performed using QIIME2 (version 2022.8), including evaluation of alpha and beta diversity, differential abundance, and correlation with experimental groups. Low-abundance OTUs (<0.01% of total reads) were removed before downstream analysis.

### Hematoxylin and eosin (H&E) staining

Histopathological evaluation was conducted in accordance with the protocol established by the reference [27], with minor modifications. In summary, following a 24-hour fixation period, the colonic tissues underwent a sequential dehydration process utilizing 70, 80, 90, 95, and 100% ethanol, followed by a 1:1 mixture of alcohol, benzene, xylene I, xylene II, paraffin I, paraffin II, and paraffin III, each for a duration of 0.5 hours. The tissue sections were subsequently dewaxed using xylene I and II for a period of 10 minutes, and then rehydrated with 100% alcohol (I, II), 90, 80, and 70% alcohol for 5 minutes, stained with hematoxylin, differentiated using 5% acetic acid for 1 minute and reverted to blue, followed by eosin staining for 1 minute, and dehydrated through 70, 80, 90, and 100% alcohol for 10 seconds, concluding with dehydration using xylene for 1 minute. The prepared slides were then dried within a fume hood and subsequently sealed with neutral gum. The morphological alterations in the tissues were examined under a light microscope (108–6290, Nikon Corporation, Tokyo, Japan), with photographic documentation captured at a magnification of 100 ×.

### Immunohistochemistry (IHC)

Immunohistochemistry was employed to measure the expression of different proteins and immune markers, including MUC2 (1:2000), NLRP3 (1:1000), cleaved caspase 3 (1:500), and CHOP (1:500) in the colon tissues of mice. Paraffin-embedded colon tissue sections were deparaffinized in xylene and incubated with 3% H2O2 to block endogenous peroxidase activity. The tissue sections were treated for antigen retrieval by heating in citrate buffer in a microwave for 10 minutes, followed by cooling at room temperature. Following a blocking step with normal goat serum, the sections were incubated with primary antibodies at 4 °C overnight and subsequently counterstained with DAPI. Protein expression levels were examined and analyzed by optical microscopy, and images were captured at 100x magnification.

### C-QTOF-MS/MS analysis

The characteristic constituents of HLWDD were analyzed using an Agilent LC/MS Q-TOF system (1290-6546, USA). Chromatographic separation was performed on a Zorbax Eclipse Plus C18 column (3.0 mm × 150 mm, 1.8 μm) maintained at 30 °C, with a mobile phase consisting of 0.1% formic acid in water (phase A) and acetonitrile (phase B). The gradient elution was as follows: 5% B from 0–2 min, increasing to 35% B from 2–20 min, 50% B from 20–24 min, decreasing to 40% B from 24–25 min, rising to 95% B from 25–30 min, and maintaining 95% B from 30–35 min. The flow rate was 0.5 mL/min, and the injection volume was 0.5 μL. The Agilent G6545 Q-TOF mass spectrometer equipped with an Agilent Jet Stream Electrospray source was operated in both positive and negative ion modes. Key instrument parameters were: capillary voltage 4000 V (+)/3500 V (-), nitrogen drying gas 8 L/min at 300 °C, sheath gas 350 °C, capillary voltage 130 V, and nebulizer pressure 45 psi. The mass scan range was m/z 100–1700 at a scan rate of 8 spectra/s. Compound identification was performed based on accurate mass measurements, MS/MS fragmentation patterns, and comparison with reference standards when available. Peaks were further annotated by matching MS/MS spectra with public and in-house databases for flavonoids, saponins, and other known HLWDD constituents. Only compounds with mass errors <5 ppm and consistent fragmentation patterns were considered confidently identified.

### Statistical analysis

Data are presented as mean ± standard error of the mean (SEM). For physiological and molecular data, normality was assessed using the Shapiro-Wilk test, and homogeneity of variances was tested using Levene’s test. Normally distributed data with equal variances were analyzed by one-way ANOVA followed by Tukey’s post hoc test, while non-normally distributed data were analyzed using the Kruskal-Wallis test with Dunn’s post hoc comparisons. Biological replicates (“n”) for each group are indicated in the figure legends. Outliers were identified using Grubbs’ test and excluded only if they were due to technical errors. For microbiome analyses, alpha diversity indices (Shannon, Chao1) were compared using the Kruskal-Wallis test. Beta diversity (Bray-Curtis distance) was assessed using principal coordinates analysis (PCoA), and statistical significance between groups was determined by PERMANOVA. Differential abundance of taxa was tested using the DESeq2 method, and p-values were adjusted for multiple comparisons using the Benjamini-Hochberg false discovery rate (FDR). A p-value < 0.05 was considered statistically significant.

## Results

### HLWDD alleviated IBD induced by DSS in mice

To explore the treatment effect of HLWDD in IBD, a colitis mouse model was induced using 2.25% DSS. The model group showed pronounced symptoms, such as considerable rectal bleeding, weight loss, and diarrhea, in contrast to the healthy control group. On the other hand, these symptoms were notably alleviated in the HLWDD-treated groups following treatment (Fig. 1B, C). The disease activity index, a critical measure reflecting diarrhea, weight loss, and rectal bleeding, was sustainably higher in the DSS-treated group (*p < 0.01*), while significantly reduced in the HLWDD-treated group *(p< 0.01)* (Fig. 1D). Furthermore, HLWDD treatments led to a marked increase in colon length in the DSS-induced Ulcerative colitis mice *(p < 0.01),* outperforming the standard treatment, SASP, underscoring HLWDD’s enhanced efficacy in treating IBD (Fig. 1E, F).

### HLWDD enhanced the intestinal barrier function

**Fig. 2.**
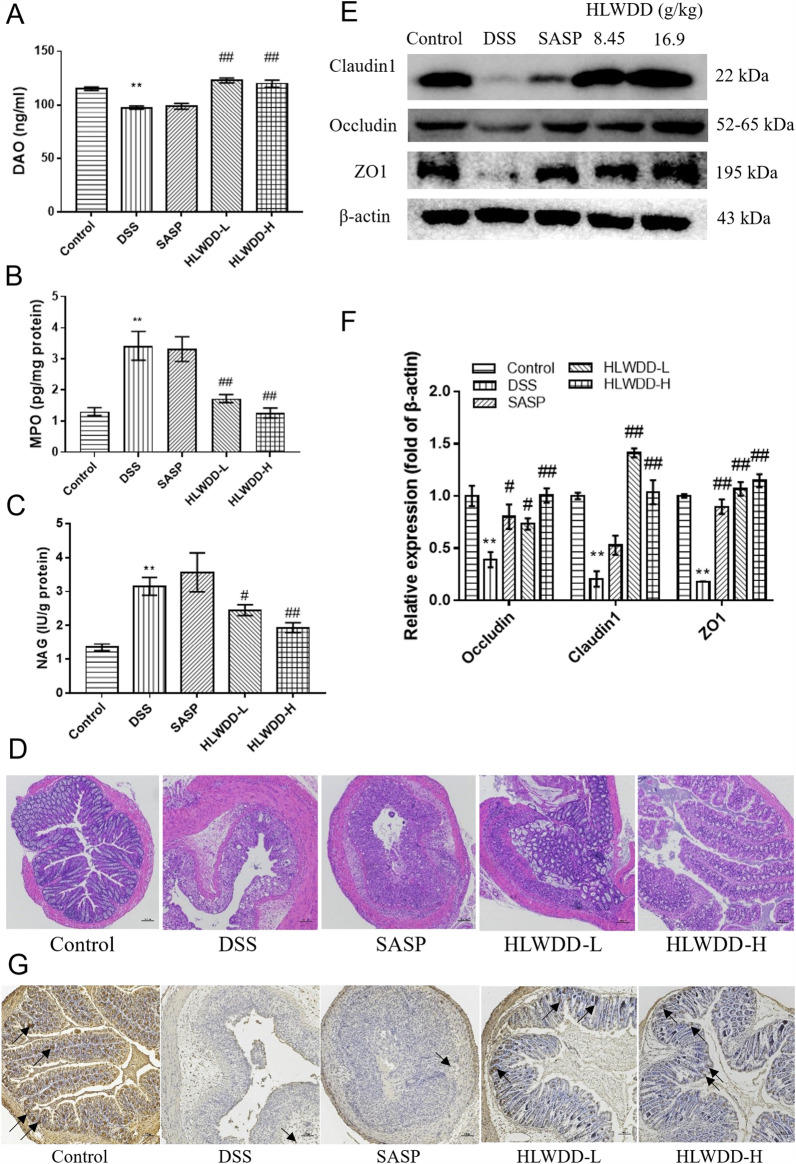
Huanglian-Wendan Decoction (HLWDD) improved the intestinal barrier function of IBD mice. **A** Effect of HLWDD on DAO level in the serum of IBD mice. Effect of HLWDD on the levels of MPO **(B)** and NAG **C** in the colon of IBD mice. **D** Histological analysis of colon tissues of IBD mice by H&E staining (magnification, 100 ×). **E** The expressions of tight junction protein ZO1, Claudin1, and Occludin were evaluated by Western Blot for the colon tissues of IBD mice. **F** Quantitative analysis for Western Blot results (n=3). **G** The expression of MUC2 was detected by IHC for the colon tissues of IBD mice (magnification, 100 ×). Data are represented as mean ± SEM, and statistical significance was analyzed by one-way ANOVA. ^*^p < 0.05, ^**^p < 0.01, vs. the control group; ^#^*p* < 0.05, ^##^*p* < 0.01, vs. the model group (DSS only)

The intestinal barrier is the critical structure to prevent the infiltration of pathogenic substances, and the indicators of DAO, MPO, and NAG reflect the colonic integrity and injury [28]. DSS, a polyanionic derivative, would destroy the intestinal epithelial cells and damage the intestinal barrier function to induce the IBD model. In this study, the level of DAO significantly decreased, while MPO and NAG increased *(p < 0.01)*, in the DSS group, reflecting the intestinal mucosal injury and the onset of gut inflammation. Following HLWDD treatment, particularly high dose, the levels of DAO, MPO, and NAG were significantly reversed *(p < 0.01)* (Fig. 2A–C). Moreover, the results of colonic histomorphology showed that the intestinal epithelial cells in the model group were sharply disordered in shape, with unclear edges, few goblet cells, and the thickening of the intestinal mucosa, indicating an increased gut permeability (compared with the control group), while those of the HLWDD-treated groups were alleviated **(**Fig. 2D). The effect of HLWDD on the tight junction proteins in IBD mice was further assessed via Western Blot and IHC assays. As a result, the expressions of Claudin1, Occludin, and ZO-1 in the DSS-treated group were significantly decreased *(p < 0.01)*, while those of the HLWDD-treated groups were significantly increased *(p < 0.01)* (Fig. 2E–F). In addition, the reduced level of Mucin-2 in the colon tissues induced by DSS was increased in the HLWDD-treated groups, indicating that HLWDD had a good potency in maintaining intestinal homeostasis (Fig. 2G). Taken together, these findings reveal that HLWDD effectively improves gut barrier integrity in IBD models.

### HLWDD inhibited the inflammation of IBD mice by suppressing TLR4/MyD88/NF-κB and IL6/JAK2/STAT3 pathways

**Fig. 3.**
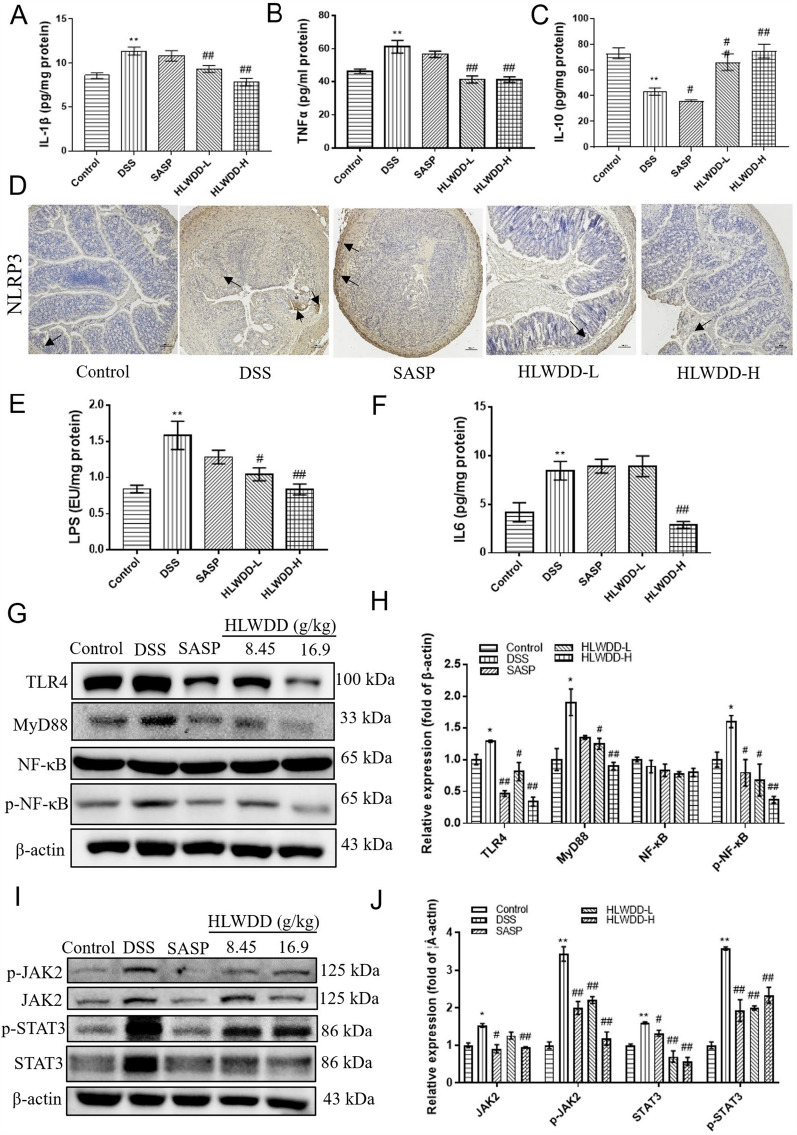
Huanglian-Wendan Decoction (HLWDD) inhibited the colonic inflammation of IBD mice by suppressing TLR4/MyD88/NF-κB and IL6/JAK2/STAT3 pathways. Effect of HLWDD on the levels of IL-1β (**A**), TNFα (**B**), and IL-10 **C** in the colon of IBD mice. **D** The expression of NLRP3 was detected by IHC assay for the colon tissues of IBD mice. Effect of HLWDD on the levels of LPS (**E**) and IL6 (**F**) in the colons of IBD mice. The expressions of the TLR4/MyD88/NF-κB pathway were evaluated by Western Blot (**G**) and quantitatively analyzed (**H**) for the colon tissues of IBD mice (n=3). The expressions of the IL6/JAK2/STAT pathway were evaluated by Western Blot (**I**) and quantitatively analyzed (**J**) for the colon tissues of IBD mice (n=3). Data are represented as mean ± SEM, and statistical significance was analyzed by one-way ANOVA. ^*^p < 0.05, ^**^p < 0.01, vs. the control group; ^#^*p* < 0.05, ^##^*p* < 0.01, vs. the model group (DSS only)

Inflammation is one of the typical characteristics of IBD [29]. In this study, the levels of IL-1β (Fig. 3A) and TNFα** (**Fig. 3B) notably increased *(p < 0.01)*, and the level of IL-10 **(**Fig. 3C) demonstrated reduced levels in the DSS group (compared with the control group, *p* < 0.05), while after HLWDD treatment, the levels of IL-1β, TNF-α, and IL-10 were significantly reversed *(p < 0.01)*. NLRP3, a key factor associated with inflammatory injury, triggers the release of pro-inflammatory cytokines like IL-1β and IL-18 upon activation. It plays a crucial role in maintaining host defense mechanisms and intestinal homeostasis [30]. DSS treatment induced a pronounced increase in NLRP3 expression, which was substantially reduced in HLWDD-treated groups **(**Fig. 3D**)**, suggesting that HLWDD could strongly suppress NLRP3 activation and reduce the production of inflammatory factors.

Intestinal damage compromises the integrity of the colonic barrier, leading to increased permeability and facilitating the infiltration of harmful substances, such as lipopolysaccharides (LPS). This infiltration activates the TLR4/MyD88/NF-κB signaling pathway, perpetuating inflammation and contributing to the progression of intestinal disorders [31]. The accumulation of harmful substances exacerbates inflammatory infiltration, further aggravating intestinal injury. Interleukin-6 (IL-6), a well-established pro-inflammatory cytokine, plays a pivotal role in driving and sustaining the inflammatory processes throughout the progression of inflammatory bowel disease (IBD) [32]. In this study, the levels of LPS and IL-6 in the colon tissues were analyzed by ELISA. Consequently, DSS treatment led to elevated levels of LPS and IL-6 *(p < 0.01)* whereas HLWDD high doses effectively attenuated these changes (*p < 0.01)* (Fig. 3E–F**)**. Furthermore, Western blotting analysis confirmed that DSS markedly upregulated the levels of TLR4, MyD88, phospho-NF-κB, phospho-JAK2, phospho-STAT3, JAK2, and STAT3. HLWDD effectively counteracted these effects, whereas total NF-κB protein levels showed no statistically significant change based on densitometric quantification (Fig. 3G–J**)**. Together, these data revealed that HLWDD showed a good effect on inhibiting inflammation by suppressing TLR4/MyD88/NF-κB and IL-6/JAK2/STAT3 pathways.

### HLWDD alleviates IBD by suppressing apoptosis and endoplasmic reticulum stress

**Fig 4.**
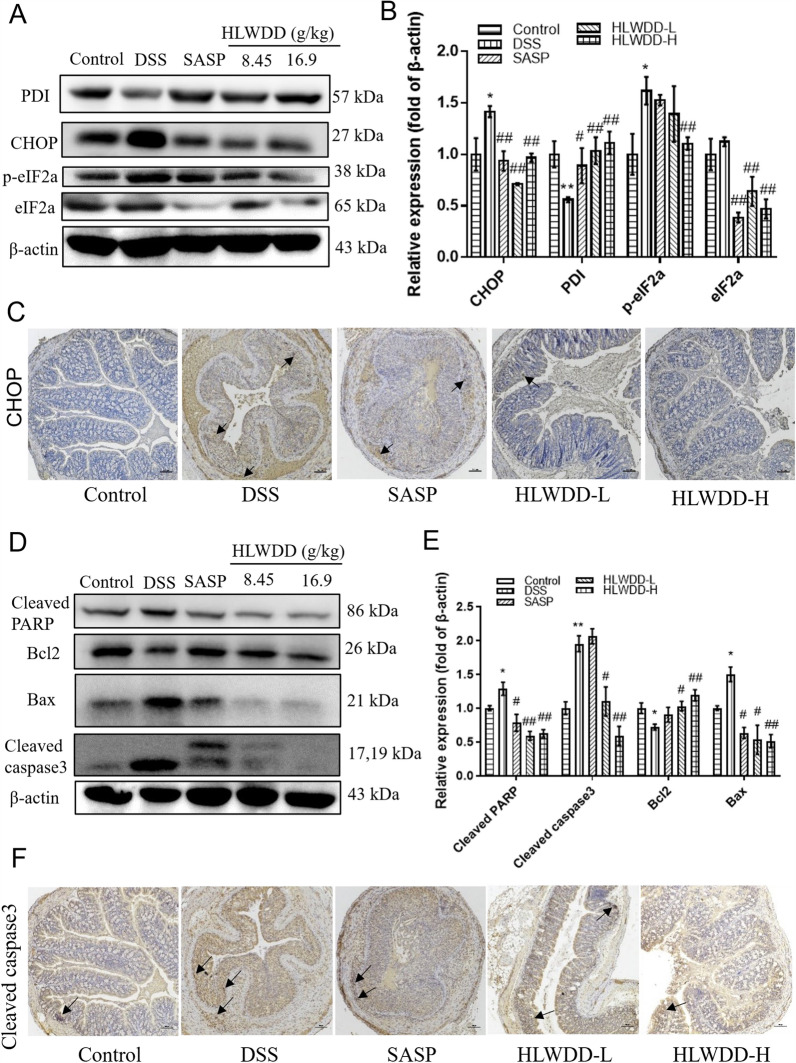
HLWDD alleviates DSS-induced IBD by suppressing endoplasmic reticulum stress and apoptosis. **A–B** Western blot analysis showing increased CHOP, phospho-eIF2α, and total eIF2α, and decreased PDI in DSS-treated mice, which were reversed after HLWDD treatment. **C** Immunohistochemical staining confirming modulation of ERS-related proteins in colon tissues. **D–F** HLWDD decreased pro-apoptotic proteins (Bax, cleaved PARP, cleaved-caspase 3) and increased the anti-apoptotic protein Bcl-2, indicating inhibition of apoptosis. Data are presented as mean ± SEM. *p < 0.05, **p < 0.01 vs. DSS group

Endoplasmic reticulum stress (ERS) is critical to maintaining intracellular calcium and protein homeostasis, and a previous study indicated that it might be a potential strategy for treating IBD by alleviating ERS [10]. In the present study, the protein expressions of CHOP, phosphor-eIF2α, and eIF2α in the DSS-induced colitis led to increased expression of CHOP *(p < 0.05), phospho-eIF2α (p < 0.01)* and total eIF2α, accompanied by a reduction in PDI *(p < 0.01)*, indicating the activation of ER stress in the IBD model. Following HLWDD treatment, the levels of CHOP, phospho-eIF2α, and eIF2α were significantly reduced *(p < 0.01)*, while PDI expression was restored (Fig. 4A–B), which was consistent with the result of the IHC assay (Fig. 4C). Apoptosis, another important pathological mechanism of IBD, would increase the intestinal permeability of intestinal epithelial cells [11]. The study revealed that continuous and severe ERS could induce apoptosis of intestinal epithelial cells, eventually increasing intestinal permeability and intensifying the inflammatory response [33]. The results of this study showed that HLWDD significantly increased the expression of Bcl-2 *(p < 0.01)*, while decreasing the expression of cleaved PARP, Bax, and cleaved caspase 3 in the colon tissues of IBD mice *(p < 0.01)* (Fig. 4D–F), indicating a good inhibitory effect of HLWDD on apoptosis. Taken together, HLWDD demonstrated good efficiency in alleviating IBD through the suppression of apoptosis and endoplasmic reticulum stress.

### HLWDD improved the gut microbiota structure and diversity of IBD mice

**Fig. 5.**
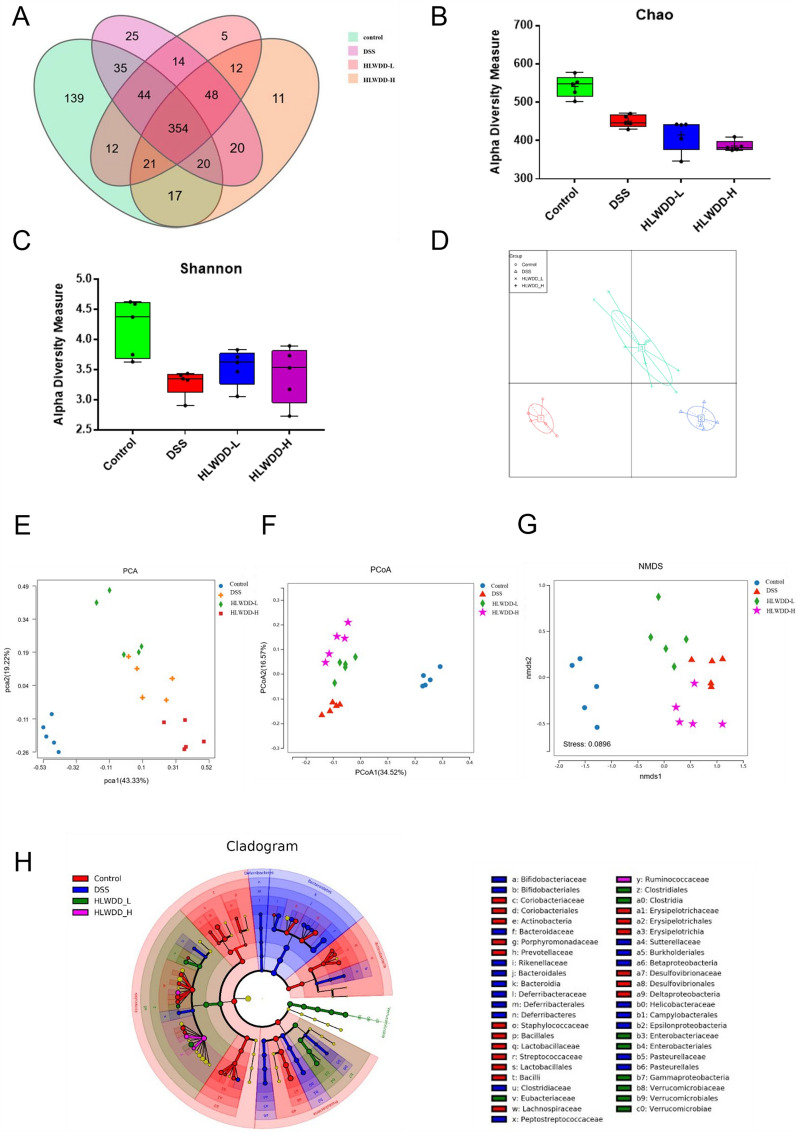
Huanglian-Wendan Decoction (HLWDD) treatment altered the gut microbiota diversity and structure in IBD mice. **A** Venn diagram showing shared OTUs among different groups. Alpha diversity indices, including Chao (**B**) and Shannon (**C**), were used to assess species richness and diversity. **D** Bacterial community composition analysis. β-diversity of microbial communities was evaluated based on unweighted UniFrac distances using principal coordinate analysis (PCoA) as the primary visualization method (**E**) and non-metric multidimensional scaling (NMDS) (**F**), while principal component analysis (PCA) **G** was additionally applied to confirm the consistency of community clustering patterns. **H** Cladogram showing differential microbial taxa among the control, DSS, HLWDD-L, and HLWDD-H groups

The gut microbial community plays a central role in modulating inflammation and immunity, significantly influencing the progression of IBD. To investigate whether the therapeutic effects of HLWDD in IBD are associated with modulation of gut microbiota, high-throughput 16S rRNA gene sequencing was performed on fecal bacterial DNA from mice. Analysis of Operational Taxonomic Units (OTUs) revealed that the control, DSS, HLWDD-L, and HLWDD-H groups shared 354 common OTUs, while each group also exhibited distinct OTUs. Notably, the control group shared more OTUs with the HLWDD-treated groups (HLWDD-L and HLWDD-H) than with the DSS group, indicating that HLWDD treatment shifted the microbial composition toward that of the control group. Each group also exhibited distinct OTUs, with 139, 25, 5, and 11 unique OTUs in the control, DSS, HLWDD-L, and HLWDD-H groups, respectively, highlighting group-specific microbial features (Fig. 5A**)**. The relatively lower number of unique OTUs observed in the HLWDD-treated groups compared with the control group may reflect a shift toward a more stable microbial community structure rather than a simple increase in richness. This observation should be interpreted together with the alpha diversity indices (Chao and Shannon), which provide a more comprehensive evaluation of microbial richness and diversity.

To assess microbial diversity within groups, alpha diversity indices were calculated, including species richness (Chao index) and overall diversity (Shannon index). The results showed that the Chao index was lower in the HLWDD-treated groups compared with the DSS group, indicating a reduction in total species richness. However, the Shannon index was significantly higher, suggesting an increase in microbial diversity. This pattern indicates that although the total number of taxa decreased, the distribution of microbial species became more even, reflecting a reduction in the dominance of certain taxa and a more balanced microbial community structure in the HLWDD-treated groups **(**Fig. 5B, C**)**. To further examine differences in microbial community structure among groups, beta diversity analysis was performed based on unweighted UniFrac distances. Principal coordinate analysis (PCoA) was used as the primary method to visualize the separation of microbial communities among groups. In addition, principal component analysis (PCA) and non-metric multidimensional scaling (NMDS) were performed to confirm the robustness of the observed clustering patterns. These analyses consistently demonstrated distinct clustering of bacterial communities among the groups, with HLWDD-treated groups showing a compositional shift toward the control group compared with the DSS group **(**Fig. 5D–G**)**. Further confirmation was provided by the cladogram analysis, which illustrated marked variations in microbial composition between the DSS group and HLWDD-treated groups, emphasizing the impact of HLWDD on restoring gut microbial composition and mitigating dysbiosis **(**Fig. 5H**)**. These findings strongly suggest that the therapeutic effects of HLWDD in IBD are closely linked with its ability to regulate gut microbiota composition and diversity.

### HLWDD modulates specific gut microbial taxa and associated SCFA production in IBD mice

**Fig. 6.**
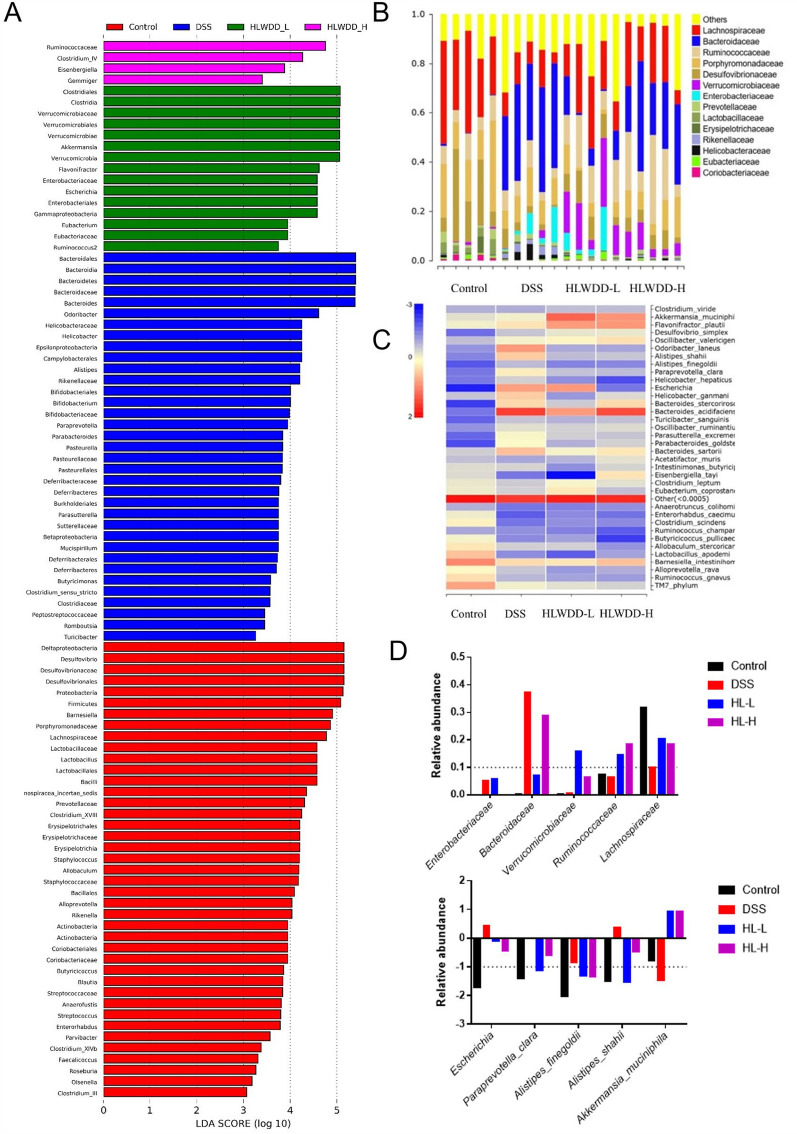
Huanglian-Wendan Decoction (HLWDD) changed the composition of gut microbiota. **A** Distribution histograms of control, DSS, HLWDD-L, and HLWDD-H groups. The relative abundance of the gut microbiota at the family level **B** and comparison heatmap at the species level **(C)**. **D** The relative abundance of *Enterobacteriaceae*, *Bacteroidaceae*, *Verrucomicrobiaceae*, *Ruminococcaceae*, *Lachnospiraceae*, *Escherichia*, *Paraprevotella_clara*, *Alistipes_finegoldii*, *Alistipes_shahii,* and *Akkermansia_muciniphila*

The linear discriminant analysis (LDA) of effect size (LEfSe) was performed to identify specific gut microbiota alterations induced by HLWDD treatment in IBD mice. As illustrated in Fig. 6A, noteworthy variations were observed in the predominance of bacterial communities across the four groups at various phylogenetic levels. A histogram at the family level and a comparison heatmap at the species level revealed notable trends in gut microbiota composition. Pathogenic bacteria, including *Enterobacteriaceae, Bacteroidaceae*, *Escherichia, Paraprevotella*, and *Alistipes,* are associated with intestinal dysbiosis and inflammatory responses [33, 34] and exhibited a relative enrichment in the DSS group. However, these pathogenic bacteria were significantly reduced in the HLWDD-treated groups. Additionally, compared to the DSS group, the relative abundance of *Verrucomicrobiaceae, Ruminococcaceae, Lachnospiraceae,* and *Akkermansia*, all of which are beneficial commensal bacteria that perform essential functions in maintaining gut homeostasis, was significantly increased after HLWDD treatment (Fig. 6B–D). These results indicate that HLWDD significantly altered the microbial composition, reducing putative pathobionts and increasing beneficial taxa.

The 16S rRNA sequencing analysis revealed that the HLWDD-treated groups exhibited a significant predominance of *Ruminococcaceae*, *Lachnospiraceae*, and *Akkermansia*. These beneficial genera are known for their roles in enhancing short-chain fatty acid (SCFA) metabolism, which supports gut barrier integrity, regulates immune responses, and maintains intestinal homeostasis. This microbial shift suggests that the changes induced by HLWDD may contribute to increased SCFA levels, rather than directly providing stimulation [35, 36]. It is well-established that short-chain fatty acids (SCFAs) play a crucial role in maintaining the normal function of the intestine and preserving the morphology of colonic epithelial cells [37].To further investigate whether HLWDD modulates SCFA metabolism in intestinal bacteria, the levels of SCFAs in fecal samples from each group of mice were measured using LC-MS/MS [38]. Our results demonstrated that the levels of acetate, butyrate, isobutyrate, and total SCFAs were significantly higher in the HLWDD-H-treated group compared to the DSS group. However, no significant differences were observed in the levels of caproate, isovalerate, propionate, and valerate between the groups. Thus, the increase in SCFAs after HLWDD treatment was mainly limited to acetate, butyrate, and isobutyrate, which corresponded with the observed shifts in the gut microbial community profile **(Fig. S1)**.

### Antibiotic pre-treatment abolished the efficacy of HLWDD against IBD

**Fig. 7.**
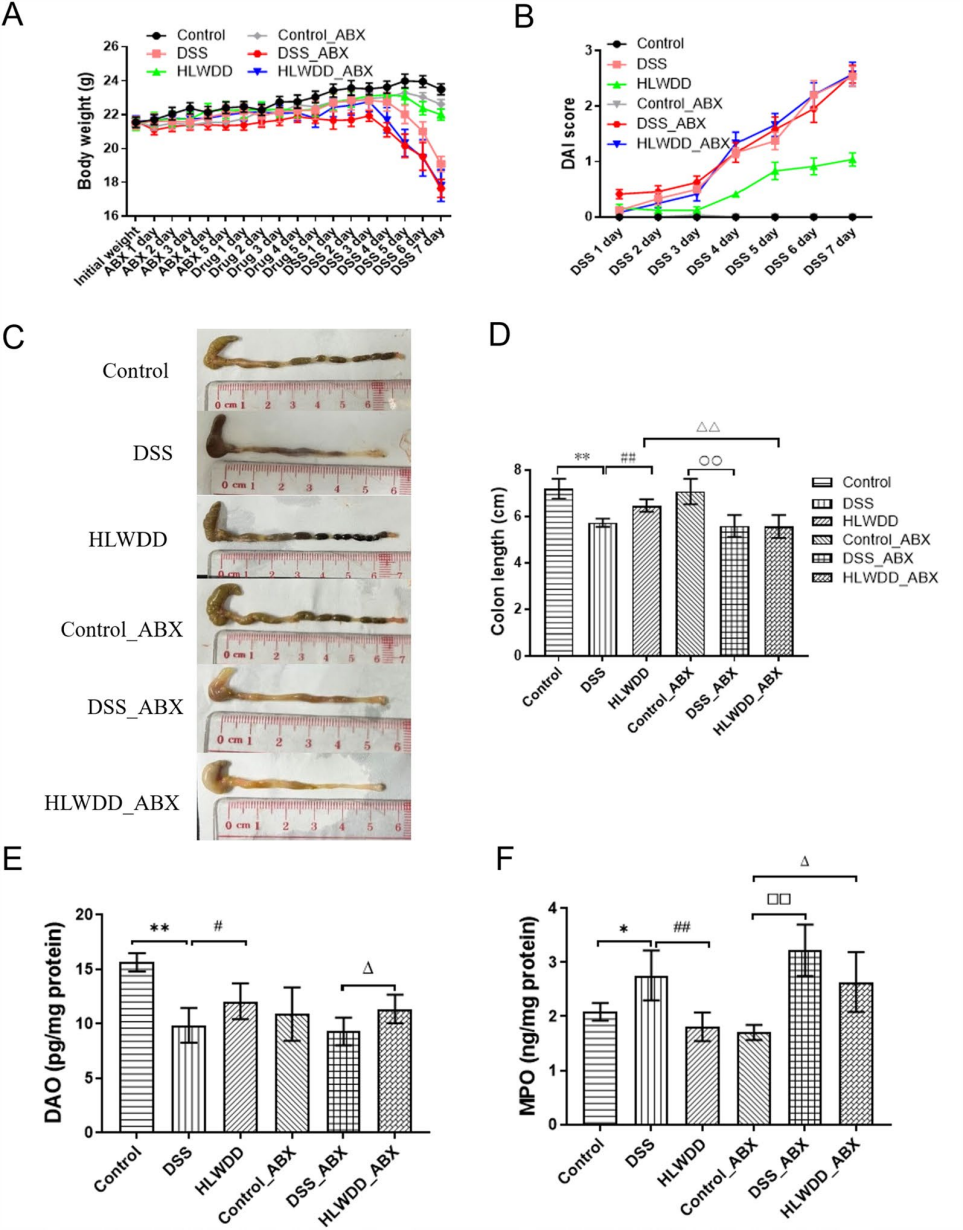
Antibiotics pre-treatment abolished the efficacy of Huanglian-Wendan Decoction (HLWDD) in treating IBD. **A** Effect of HLWDD with or without ABX on the body weight of IBD mice. **B** Disease activity index of IBD mice. **C** Representative photographs of the colon. **D** Effect of HLWDD with or without ABX on the colon length of IBD mice. Effect of HLWDD with or without ABX on the levels of DAO (**E**) and MPO (**F**) in the colon of IBD mice. Data are represented as mean ± SEM, and statistical significance was analyzed by one-way ANOVA, ^*^p < 0.05, ^**^p < 0.01, vs. the control group; ^#^*p* < 0.05, ^##^*p* < 0.01, vs. the model group (DSS only)

To further investigate the potential remedial role of HLWDD on IBD, which is dependent on the gut microbiota, mice were pre-treated with antibiotics (ABX) to deplete their gut microbiota. The alleviated effects of HLWDD on body weight loss **(**Fig. 7A**)**, DAI score** (**Fig. 7B**)**, and colon length **(**Fig. 7C–D**)** were significantly abolished by the antibiotic pre-treatment, suggesting that HLWDD’s protective effects against IBD are indeed microbiota-dependent. However, the levels of DAO** (**Fig. 7E**)** and MPO **(**Fig. 7F**)** were not entirely reversed by ABX treatment, indicating that HLWDD may exert its therapeutic effects through additional mechanisms beyond modulation of the gut microbiota.

### Antibiotics pre-treatment depleted the alleviation of HLWDD on the injury of the gut barrier

**Fig. 8.**
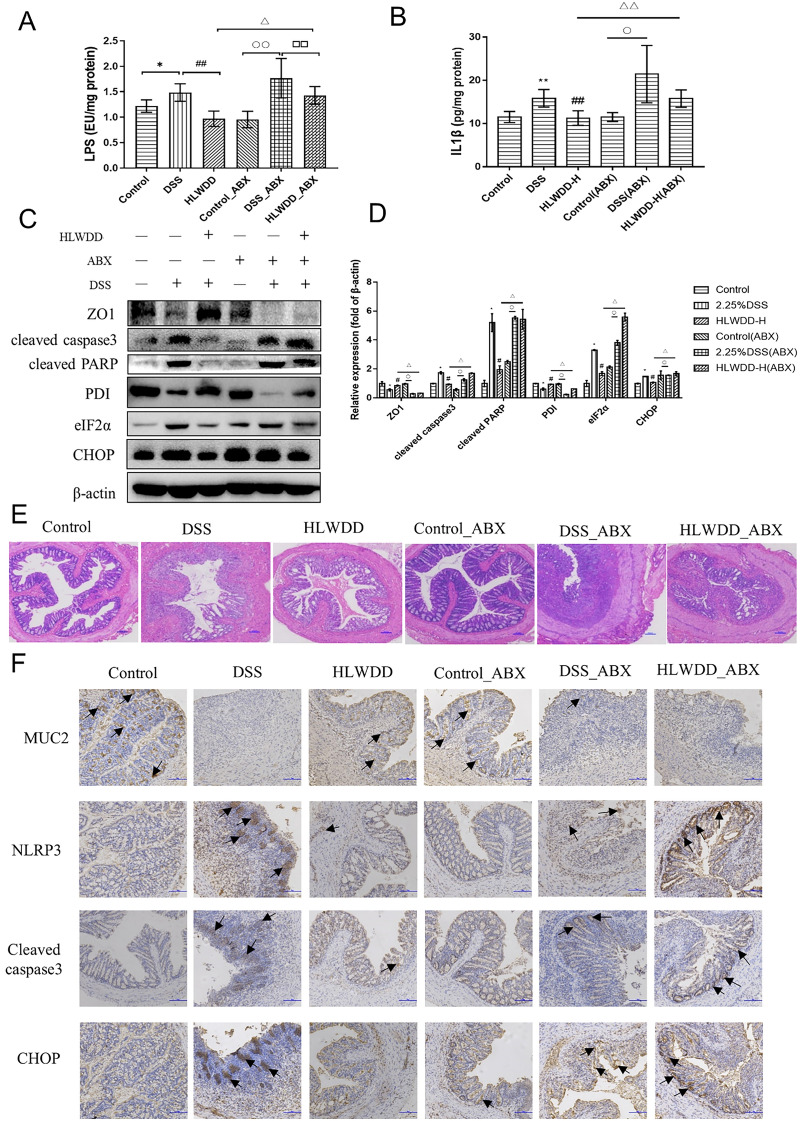
Antibiotic pre-treatment depleted the alleviation of Huanglian-Wendan Decoction (HLWDD) on the injury of the gut barrier. Effect of HLWDD with or without ABX on the levels of LPS (**A**) and IL-1β (**B**)**.** The expression of ZO1 in the colon tissues of IBD mice was evaluated by Western Blot (**C**) and quantitatively analyzed (**D**) for the colons of IBD mice (n=3). **E** Histological analysis of colon tissues by H&E staining. **F** The expressions of MUC2, NLRP3, CHOP, and cleaved caspase-3 were detected by IHC for the colon tissues of IBD mice. Data are represented as mean ± SEM, and statistical significance was analyzed by one-way ANOVA, ^*^p < 0.05, ^**^p < 0.01, vs. the control group; ^#^*p* < 0.05, ^##^*p* < 0.01, vs. the model group (DSS only)

The impact of antibiotic (ABX) treatment on the ability of HLWDD to inhibit inflammation and restore intestinal barrier function in IBD mice was also assessed. The results demonstrated that the levels of LPS and IL-1β in the HLWDD_ABX group were markedly elevated compared to the HLWDD group (Fig. 8A, B). HLWDD treatment significantly upregulated ZO-1 expression in colon tissues, whereas this effect was dramatically diminished in the HLWDD_ABX group **(**Fig. 8C, D**)**. Additionally, histological analysis through H&E staining and IHC assays revealed that ABX treatment reversed the beneficial effects of HLWDD, including the restoration of intestinal structural integrity, upregulation of Mucin-2 expression, and downregulation of NLRP3, cleaved caspase 3, and CHOP expression **(**Fig. 8E, F**)**. These findings suggest that the therapeutic effects of HLWDD on IBD and intestinal barrier restoration are, at least in part, dependent on the gut microbiota, as ABX treatment markedly attenuated these benefits.

### Identification of the phytochemicals of HLWDD

**Fig. 9.**
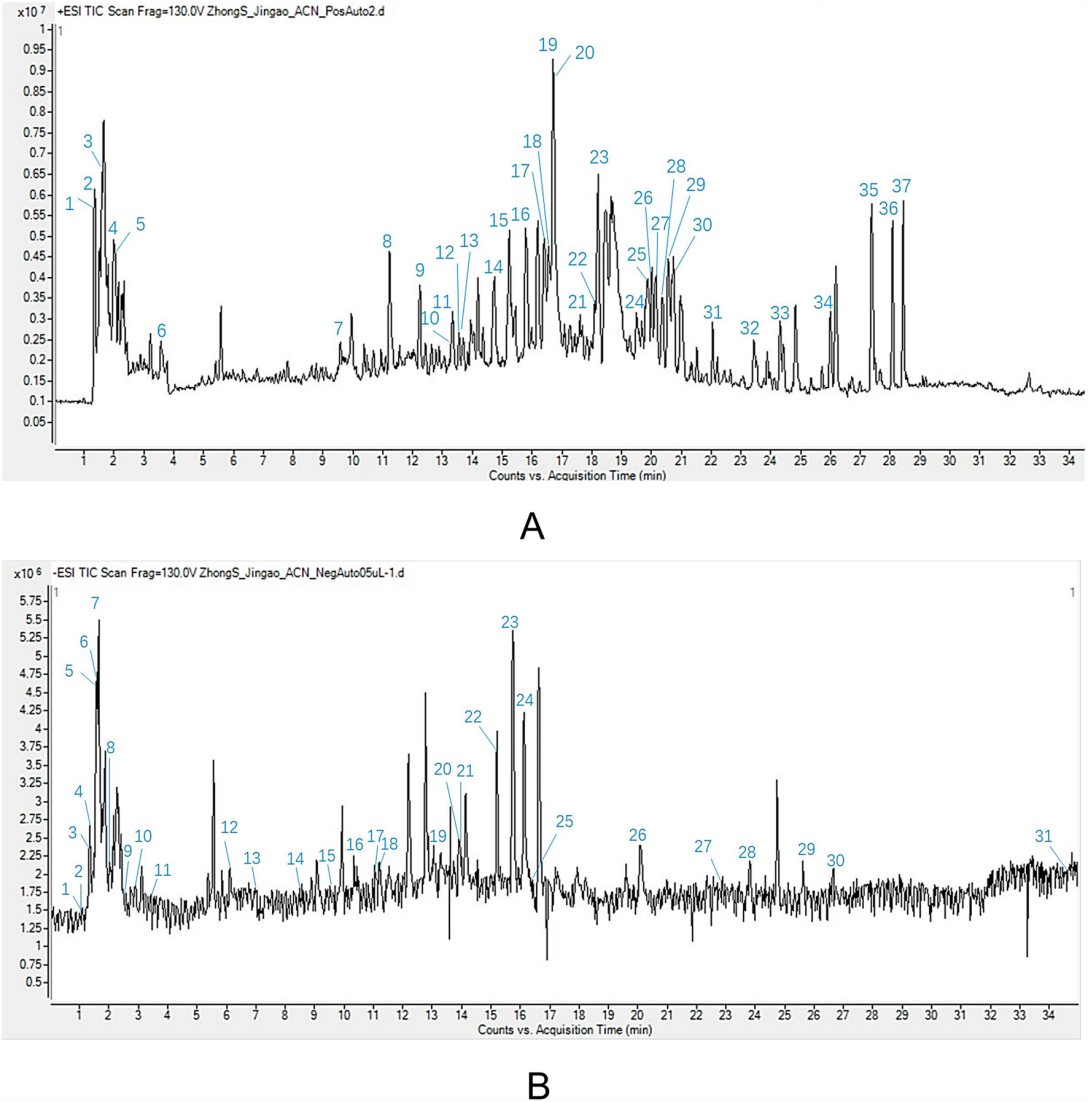
Identification of phytochemicals in Huanglian-Wendan Decoction (HLWDD) in positive mode (**A**) and negative mode (**B**)

The chemical composition of HLWDD was analyzed using LC-QTOF-MS/MS in both positive and negative ion modes. In the positive mode, 37 major compounds were identified, including alkaloids, flavonoids, and organic acids** (**Fig. 9A**)**. Notable compounds include betaine, dihydromyricetin, synephrine, cularine, glaucine, epiberberine, hesperetin, coptisine, and palmatine. In the negative mode, 31 major compounds were detected, comprising phenolic acids, organic acids, and flavonoids **(**Fig. 9B**)**. Key identified metabolites include lactic acid, fumaric acid, succinic acid, protocatechuic acid, papaverine, glycyrrhizic acid, and drummondin A. The detailed mass spectral data and structural information of these compounds are summarized in Tables 2 and 3.

**Table 2:** Components identified in HLWDD by LC-QTOF-MS-MS in the positive ion mode

Peak	RT	Mass	Name	DB formula	DB diff (ppm)
1	1.58	117.0787	Betaine	C5H11NO2	2.63
2	1.645	129.0795	6xi-methoxypiperidin-2-one	C6H11NO2	− 4.4
3	1.673	143.0954	Dihydromyricetin	C15H12O8	12366423
4	1.999	149.084	D-Cathinone	C9H11N	0.28
5	2.001	167.0943	Synephrine	C9H13NO2	1.86
6	3.618	179.0948	N-methylphenylalanine	C10H13NO2	− 0.76
7	9.579	313.1684	3-Epischelhammericine	C19H23NO3	− 1.81
8	11.235	341.1628	Cularine	C20H23NO4	− 0.25
9	12.251	368.1115	3-O-Feruloylquinic acid	C17H20O9	− 2.15
10	13.3	335.0792	Nandazurine	C19H13NO5	0.42
11	13.359	339.1475	O-methylbulbocapnine	C20H21NO4	− 1.39
12	13.562	351.1111	Oxyberberine	C20H17NO5	− 1.09
13	13.697	355.1784	Glaucine	C21H25NO4	− 0.18
14	14.763	323.1158	Rutacridone epoxide	C19H17NO4	− 0.09
15	15.253	580.1795	Narirutin	C27H32O14	− 0.49
16	15.799	272.0692	Aromadendrin-5,7- dimethyl ether	C15H12O5	− 2.53
17	16.573	336.1241	Epiberberine	C20H18NO4	− 1.4
18	16.696	302.0794	Hesperetin	C16H14O6	− 1.03
19	16.704	337.128	Jatrorrhizine	C20H19NO4	10.16
20	16.783	320.0927	Coptisine	C19H15ClNO4	112393.14
21	17.53	367.1427	Corynoline	C21H21NO5	− 1.9
22	18.091	333.1011	Dihydrosanguinarine	C20H15NO4	− 2.99
23	18.476	351.15	Palmatine	C21H21NO4	− 8.41
24	19.509	365.1631	Tylophorinidine	C22H23NO4	− 1.14
25	19.885	439.143		C28H17N5O	
26	20.007	349.1323	Dihydrochelerythrine	C21H19NO4	− 2.5
27	20.033	469.153		C28H23NO6	
28	20.132	469.1535		C29H19N5O2	
29	20.373	455.1739		C28H25NO5	
30	20.706	485.184		C29H27NO6	
31	22.056	724.2211	Arillanin A	C33H40O18	0.49
32	23.439	242.0947	Cearoin	C15H14O3	− 1.68
33	24.419	372.1208	Isosinensetin	C20H20O7	0.18
34	26.182	260.1052	Hemigossypol	C15H16O4	− 1.3
35	27.386	402.1316	(3'R,4'R)−3'- Epoxyangeloyloxy-4'- acetoxy-3',4'- dihydroseselin	C21H22O8	− 0.36
36	28.069	432.1425	3,3',4',5,5',6,7-Heptamethoxyflavone	C22H24O9	− 1.14
37	28.428	372.1214	5,7,2',4',6'-Pentamethoxyflavone	C20H20O7	− 1.27

**Table 3:** Components identified in HLWDD by LC-QTOF-MS-MS in the negative ion mode

Peak	RT	Mass	Name	DB formula	DB diff
1	1.556	332.0846	1-Hydroxy-2,3,4,7- tetramethoxyxanthone	C17H16O7	14.95
2	1.591	120.0416	Methyl allyl tetrasulfide	C4H8O4	5.21
3	1.612	90.0306	Lactic acid	C3H6O3	11.7
4	1.648	192.064	D-(-)-Quinic acid	C7H12O6	− 3.07
5	1.852	192.0264	Isocitric acid d	C6H8O7	3.1
6	1.861	116.0093	Fumaric acid	C4H4O4	14.15
7	1.863	134.0201	Malic acid	C4H6O5	10.52
8	2.155	291.0943	Sarmentosin epoxide	C11H17NO8	4
9	2.41	288.0831	Dianthoside	C12H16O8	5.05
10	2.887	118.0267	Succinic acid	C4H6O4	− 0.5
11	3.13	162.0521	Danshensu	C6H10O5	4.69
12	6.113	198.0522	Salvianic acid A	C9H9NaO5	110993.9
13	6.955	154.0261	Protocatechuic acid	C7H6O4	3.26
14	8.556	268.0573	Homocystine	C8H16N2O4S2	− 8.12
15	9.937	368.1092	Methyl chlorogenate	C17H20O9	4.03
16	10.356	486.137	Cassigerol E	C28H22O8	− 11.34
17	11.072	594.1577	Oroxin B	C27H30O15	1.33
18	11.202	341.1612	Cularine	C20H23NO4	4.47
19	13.284	428.1676	Bruceine F	C20H28O10	1.56
20	13.915	550.1677	Angustiamarin	C26H30O13	1.65
21	13.979	604.251	9(betaH)−9-Dihydro- 19-acetoxy-10- deacetylbaccatin III	C31H40O12	1.7
22	15.2	580.18	Liquiritigenin-7,4'- diglucoside	C27H32O14	− 1.44
23	15.746	558.1329	(+)-Gallocatechin- hexacetate	C27H26O13	8.01
24	16.127	610.1898	Mesuein	C28H34O15	0
25	16.364	339.1458	Papaverine	C20H21NO4	3.72
26	20.09	594.1936	Isosakuranetin-7- rutinoside	C28H34O14	2.11
27	22.903	272.0672	Butein	C15H12O5	4.57
28	23.814	838.398	Glyyunnanprosapogeni n D	C42H62O17	0.88
29	25.629	822.4037	Glycyrrhizic acid	C42H62O16	0.12
30	26.645	470.1928	Drummondin A	C26H30O8	2.65
31	34.791	256.2393	13-Methyl pentadecanoic acid	C16H32O2	3.72

## Discussion

Huanglian-Wendan Decoction (HLWDD) has been used clinically for gastrointestinal disorders; its underlying mechanisms in inflammatory bowel disease (IBD) remain largely unknown. In this study, we provide the first evidence that HLWDD markedly ameliorates DSS-induced colitis in mice. Importantly, the protective effects of HLWDD are mediated not only through suppression of intestinal inflammation and endoplasmic reticulum (ER) stress but also through modulation of gut microbial composition, revealing a mechanism dependent on the restoration of gut microbial homeostasis. These findings are significant because dysregulation of the gut microbiota plays a central role in IBD pathogenesis, influencing immune responses, epithelial barrier integrity, and metabolic activity. By demonstrating that HLWDD simultaneously regulates inflammatory signaling, ER stress pathways, and microbial community structure, our study provides novel mechanistic insight into how this traditional herbal formula promotes intestinal homeostasis and mitigates colitis, highlighting its potential as a therapeutic strategy for IBD.

DSS administration has been shown to trigger classical IBD manifestations, including body weight loss, rectal bleeding, colon shortening, and histopathological damage [39].In agreement with these reports, our study also observed similar pathological features in DSS-treated mice; however, HLWDD treatment markedly alleviated manifestations such as reduced body weight, loose stools, bloody stools, and shortened colon length. These findings highlight HLWDD’s therapeutic potential and provide a foundation for further investigation into its underlying mechanisms in IBD.

Impaired intestinal barrier function and an ongoing inflammatory response are the hallmarks of the pathogenesis of IBD [40]. DSS would destroy the intestinal structure and alter intestinal permeability, thereby enhancing the entry of harmful substances into the intestine, leading to a continuous inflammatory response. Our study showed that the intestinal epithelial cells of mice were seriously affected after DSS induction, and the expressions of tight junction proteins remarkably decreased, resulting in the activation of inflammation responses. The TLR4/MyD88/NF-κB signaling pathway is a key mediator of innate immune responses triggered by microbial components and plays an important role in intestinal inflammation and epithelial barrier dysfunction in IBD [41]. In addition, the IL-6/JAK2/STAT3 pathway is a major cytokine-driven inflammatory signaling cascade that contributes to immune dysregulation and chronic intestinal inflammation [42]. Therefore, these two pathways were selected to investigate the potential molecular mechanisms underlying the therapeutic effects of HLWDD. LPS, a bacterial endotoxin, can activate the TLR4/MyD88/NF-κB pathway by promoting the secretion of IL-6, IL-1β, and TNF-α, thereby aggravating inflammatory responses. IL-6, mainly produced by mononuclear macrophages, Th2 cells, vascular endothelial cells, and fibroblasts, participates in the occurrence and development of inflammation and cancer, via inducing inflammation and regulating immunity [43]. It would also induce the formation of JAK2 phosphorylated dimer, which in turn induces the phosphorylated STAT3 dimer into the nucleus, and promotes the transcription of genes downstream, such as IL-1β, IL-18, and NLRP3 [44]. In this study, HLWDD significantly decreased the levels of LPS, IL6, IL-1β, and TNFα, and alleviated inflammation by suppressing TLR4/MyD88/NF-κB and IL6/JAK2/STAT3 pathways.

Endoplasmic reticulum stress (ERS) has recently emerged as a key process in the pathogenesis of IBD. The ER is involved in the regulation of cellular Ca 2+ balance and protein folding, and the disruption of its homeostasis leads to induction of the unfolded protein response (UPR), which has been linked with the development of mucosal inflammation and mucosal epithelial damage [45]. DSS-induced colitis induced ERS in our study, with upregulated CHOP and phospho-eIF2α and downregulated PDI expression of protein, as well as an increase in total eIF2α compared to control. These findings are in agreement with the reported UPR induction during DSS-mediated colitis [46]. After HLWDD treatment, CHOP, phospho-eIF2α, and eIF2α levels were significantly reduced, whereas PDI expression was restored. IHC staining confirmed the same trend, indicating that HLWDD effectively alleviates ERS in colitic tissues. Similar effects have been reported for other natural compounds such as curcumin and berberine, which also reduce ERS signaling and protect mucosal integrity [47].

The gut microbiota is a complex community of microbes that plays a vital role in digestion and metabolism, immune regulation, infection resistance, and drug metabolism [48]. Previous studies have revealed that the intestinal microbiota plays a crucial role in the onset of IBD by influencing inflammation, metabolic pathways, cellular processes, and genetic information processing [49, 50]. The gut microbiota consists of beneficial, pathogenic, and neutral bacteria. Probiotics, a key component of beneficial bacteria, perform a range of essential functions, including the production of digestive enzymes that aid in the digestion and absorption of intestinal nutrients, as well as the activation of the immune system [51], stimulating the production of secretory globulin in the intestinal tract, relieving inflammation, reducing the permeability of intestinal mucosa, inhibiting the apoptosis of epithelial cells, and protecting the intestinal barrier [52].

As a rich obligate anaerobe in the intestine, *Lachnospiraceae* can recover the hematopoietic function, repair the gastrointestinal tract, and support colonization resistance against intestinal pathogens via butyric acid production and participating in bile acid metabolism [36, 53–55]. Studies reported that *Ruminococcaceae* was regarded as a major microbe for mediating the transformation of primary into secondary bile acids and enhancing intestinal stem cell growth [56], and *Akkermansia* could relieve metabolic disorders, increase the production of propionate and acetate [57], and stimulate goblet cells to produce mucin, which strengthens the intestinal barrier to prevent bacterial imbalance [35]. A series of pernicious bacteria, such as *Enterobacteriaceae*, *Parasutterella*, and *Escherichia*, are related to IBD pathogenesis and progression [33, 58]. *Alistipes*, also known as a conditioned pathogen, has pathogenicity in colorectal cancer and promotes the proliferation of cancer cells [59]. In this study, HLWDD was found to increase the abundance of beneficial bacteria, including *Lachnospiraceae*, *Ruminococcaceae*, and *Akkermansia*, and decrease the abundance of pathogenic bacteria, such as *Enterobacteriaceae*, *Parasutterella*, and *Escherichia*. Moreover, HLWDD increased the levels of short-chain fatty acids, such as acetate, butyrate, and isobutyrate, which have been reported to play an important role in maintaining intestinal homeostasis and function [60].

The chemical composition of HLWDD was characterized using LC-QTOF-MS/MS in both positive and negative ion modes. In the positive ion mode, 37 compounds were identified, mainly including alkaloids, flavonoids, and organic acids, such as betaine, dihydromyricetin, synephrine, cularine, glaucine, epiberberine, hesperetin, coptisine, and palmatine. In the negative ion mode, 31 compounds were detected, including phenolic acids and organic acids, such as lactic acid, fumaric acid, succinic acid, protocatechuic acid, papaverine, glycyrrhizic acid, and drummondin A. Several of these identified compounds have been reported to exhibit anti-inflammatory, antioxidant, and intestinal barrier–protective activities, which may collectively contribute to the therapeutic effects observed in this study. For example, isoquinoline alkaloids such as epiberberine, coptisine, and palmatine (berberine-type alkaloids) can suppress activation of the NF-κB signaling pathway and reduce inflammatory cytokine production, thereby alleviating intestinal inflammation [61]. Flavonoids such as Hesperetin and Dihydromyricetin have been reported to attenuate oxidative stress and protect intestinal epithelial cells [62]. In addition, Glycyrrhizic acid possesses anti-inflammatory and mucosal protective effects and has been shown to improve intestinal barrier integrity [63].Phenolic acids such as Protocatechuic acid also exhibit antioxidant and anti-inflammatory activities that may contribute to the alleviation of intestinal [64]. Collectively, these compounds may act synergistically to regulate inflammatory signaling, reduce epithelial apoptosis, and alleviate Endoplasmic Reticulum Stress, thereby improving intestinal barrier function and reducing colitis severity. These findings suggest that the pharmacological effects of HLWDD are likely mediated through the combined actions of multiple bioactive constituents.

Despite the novel findings of this study, several limitations should be noted. The DSS-induced acute colitis model does not fully capture the chronic, relapsing nature of human IBD, and future studies using chronic or adoptive transfer models are warranted. While 16S rRNA sequencing revealed associations between HLWDD treatment and gut microbial shifts, causation remains unproven, and fecal microbiota transplantation experiments will be necessary to confirm microbial involvement. Although LC-MS/MS identified HLWDD constituents, the specific bioactive compounds responsible for the observed effects remain unclear. The study analyzed whole colon tissue, which does not allow determination of whether the observed STAT3, NF-κB, and ER stress changes occur in epithelial or immune cells; future cell-type–specific studies are needed. In addition, the mechanistic contribution of key signaling pathways, including TLR4, JAK2/STAT3, and NLRP3, was not directly tested with pharmacologic inhibitors or genetic tools, which should be explored in future work to validate their roles in mediating HLWDD’s effects.

## Conclusion

In conclusion, this study demonstrates that Huanglian-Wendan Decoction (HLWDD) effectively alleviates DSS-induced IBD by suppressing intestinal inflammation, improving epithelial barrier integrity, and restoring gut microbial balance. Mechanistically, HLWDD modulated key inflammatory signaling pathways and reduced endoplasmic reticulum stress–associated apoptosis while enhancing tight junction protein expression. Importantly, the loss of its protective effects following antibiotic-induced microbiota depletion highlights the critical role of gut microbiota in mediating its therapeutic action. These findings suggest that HLWDD represents a promising microbiota-targeted strategy for IBD management and warrants further clinical investigation.

## Supplementary Information

Supplementary Material 1

## Data Availability

The datasets generated during and/or analysed during the current study are available from the corresponding author on reasonable request.

## References

[1] Molodecky NA, et al. Increasing incidence and prevalence of the inflammatory bowel diseases with time, based on systematic review. Gastroenterology. 2012;142(1):46-54.e42. 22001864 10.1053/j.gastro.2011.10.001

[2] Ng SC, et al. Worldwide incidence and prevalence of inflammatory bowel disease in the 21st century: a systematic review of population-based studies. Lancet. 2017;390(10114):2769–78. 29050646 10.1016/S0140-6736(17)32448-0

[3] Kaplan GG. The global burden of IBD: from 2015 to 2025. Nat Rev Gastroenterol Hepatol. 2015;12(12):720–7. 26323879 10.1038/nrgastro.2015.150

[4] Kobayashi T, et al. Ulcerative colitis Nat Rev Dis Primers. 2020;6(1):74. 32913180 10.1038/s41572-020-0205-x

[5] Ge X, et al. The effects of dihydroartemisinin on inflammatory bowel disease-related bone loss in a rat model. Exp Biol Med. 2018;243(8):715–24. 10.1177/1535370218769420 PMC6378506 29763384

[6] Zhan X, et al. Polysaccharides from Garlic protect against liver injury in DSS-induced inflammatory bowel disease of mice via suppressing pyroptosis and oxidative damage. Oxid Med Cell Longev. 2022;2022:2042163. 36017235 10.1155/2022/2042163 PMC9398839

[7] Yamamoto-Furusho JK, Sarmiento-Aguilar A. Joint involvement in Mexican patients with ulcerative colitis: a hospital-based retrospective study. Clin Rheumatol. 2018;37(3):677–82. 28914369 10.1007/s10067-017-3834-z

[8] Siegel CA, et al. Risk of lymphoma associated with combination anti-tumor necrosis factor and immunomodulator therapy for the treatment of Crohn’s disease: a meta-analysis. Clin Gastroenterol Hepatol. 2009;7(8):874–81. 19558997 10.1016/j.cgh.2009.01.004 PMC2846413

[9] Tan Y-R, et al. The role of endoplasmic reticulum stress in regulation of intestinal barrier and inflammatory bowel disease. Exp Cell Res. 2023;424(1):113472. 36634742 10.1016/j.yexcr.2023.113472

[10] Qiao D, et al. Regulation of endoplasmic reticulum stress-autophagy: a potential therapeutic target for ulcerative colitis. Front Pharmacol. 2021;12:697360. 34588980 10.3389/fphar.2021.697360 PMC8473789

[11] Wan Y, et al. Excessive apoptosis in ulcerative colitis: crosstalk between apoptosis, ROS, ER stress, and intestinal homeostasis. Inflamm Bowel Dis. 2022;28(4):639–48. 34871402 10.1093/ibd/izab277

[12] Gu Z, et al. Tilapia head glycolipids reduce inflammation by regulating the gut microbiota in dextran sulphate sodium-induced colitis mice. Food Funct. 2020;11(4):3245–55. 32219260 10.1039/d0fo00116c

[13] Engevik MA, et al. Fusobacterium nucleatum secretes outer membrane vesicles and promotes intestinal inflammation. mBio. 2021. 10.1128/mBio.02706-20. 33653893 10.1128/mBio.02706-20 PMC8092269

[14] Yang WH, et al. Recurrent infection progressively disables host protection against intestinal inflammation. Science. 2017. 10.1126/science.aao5610. 29269445 10.1126/science.aao5610 PMC5824721

[15] Liu YJ, et al. Parthenolide ameliorates colon inflammation through regulating Treg/Th17 balance in a gut microbiota-dependent manner. Theranostics. 2020;10(12):5225–41. 32373209 10.7150/thno.43716 PMC7196297

[16] Crunkhorn S. Yeast probiotics treat IBD. Nat Rev Drug Discov. 2021;20(8):588. 34234307 10.1038/d41573-021-00116-5

[17] He F, et al. Gut microbiota modulate intestinal inflammation by endoplasmic reticulum stress-autophagy-cell death signaling axis. J Anim Sci Biotechnol. 2025;16(1):63. 40312439 10.1186/s40104-025-01196-8 PMC12046778

[18] Parada Venegas D, et al. Short chain fatty acids (SCFAs)-mediated gut epithelial and immune regulation and its relevance for inflammatory bowel diseases. Front Immunol. 2019;10:277. 30915065 10.3389/fimmu.2019.00277 PMC6421268

[19] Ju Y, Wei D. Analysis of “Preparative Qianjin Yaofang” Wendan Decoction. Shandong Zhong Yi Za Zhi. 2004;03:181–2.

[20] Zhang F, et al. Analysis on the clinical application of Huanglian Wendan Decoction in Liuyin Article Differentiation. Hunan Zhong Yi Xue Yuan Xue Bao. 2018;34(11):106–7.

[21] Hu YH, et al. Clinical observation on effect of modified huanglian wendan decoction in treating diabetic asymptomatic myocardial ischemia. Zhongguo Zhong Xi Yi Jie He Za Zhi. 2005;25(9):790–3. 16248239

[22] Li YB, et al. Protective effects of Huanglian Wendan Decoction aganist cognitive deficits and neuronal damages in rats with diabetic encephalopathy by inhibiting the release of inflammatory cytokines and repairing insulin signaling pathway in hippocampus. Chin J Nat Med. 2016;14(11):813–22. 27914525 10.1016/S1875-5364(16)30098-X

[23] Wang WGLLQMHLWDJ. Clinical observation on the therapeutic effect of Huanglian Wendan Decoction in the treatment of Ulcerative Colitis with the Damp-Heat Syndrome of the Large Intestine. Works in Evaluation and Anal of Drug-Use in Hospitals of China. 2024;24(4):455.

[24] Yue Q-Q, et al. Traditional Chinese medicine for premature ventricular contraction caused by obstructive sleep apnea: a case report and literature review. Med. 2025;104(5):e41206. 10.1097/MD.0000000000041206 PMC11789912 39889189

[25] Dai W, et al. Ficus pandurata Hance inhibits ulcerative colitis and colitis-associated secondary liver damage of mice by enhancing antioxidation activity. Oxid Med Cell Longev. 2021;2021:2617881. 34966476 10.1155/2021/2617881 PMC8710911

[26] Yang Y, et al. Cross-talk between the gut microbiota and monocyte-like macrophages mediates an inflammatory response to promote colitis-associated tumourigenesis. Gut. 2020;70(8):1495–506. 33122176 10.1136/gutjnl-2020-320777 PMC8292576

[27] Yeganeh PR, et al. Apple peel polyphenols reduce mitochondrial dysfunction in mice with DSS-induced ulcerative colitis. J Nutr Biochem. 2018;57:56–66. 29674247 10.1016/j.jnutbio.2018.03.008

[28] Zhou H, et al. Monosexual cercariae of Schistosoma japonicum infection protects against DSS-induced colitis by shifting the Th1/Th2 balance and modulating the gut microbiota. Front Microbiol. 2020;11:606605. 33469451 10.3389/fmicb.2020.606605 PMC7813680

[29] Yao D, et al. Inflammation and inflammatory cytokine contribute to the initiation and development of ulcerative colitis and its associated cancer. Inflamm Bowel Dis. 2019;25(10):1595–602. 31287863 10.1093/ibd/izz149

[30] Hu J, et al. Qingchang Huashi Formula attenuates DSS-induced colitis in mice by restoring gut microbiota-metabolism homeostasis and goblet cell function. J Ethnopharmacol. 2021;266:113394. 32941971 10.1016/j.jep.2020.113394

[31] Jin BR, et al. Rosmarinic acid represses colitis-associated colon cancer: a pivotal involvement of the TLR4-mediated NF-κB-STAT3 axis. Neoplasia. 2021;23(6):561–73. 34077834 10.1016/j.neo.2021.05.002 PMC8180929

[32] Waldner MJ, Neurath MF. Master regulator of intestinal disease: IL-6 in chronic inflammation and cancer development. Semin Immunol. 2014;26(1):75–9. 24447345 10.1016/j.smim.2013.12.003

[33] Ju T, et al. Defining the role of Parasutterella, a previously uncharacterized member of the core gut microbiota. ISME J. 2019;13(6):1520–34. 30742017 10.1038/s41396-019-0364-5 PMC6776049

[34] Lv LX, et al. Alterations and correlations of the gut microbiome, metabolism and immunity in patients with primary biliary cirrhosis. Environ Microbiol. 2016;18(7):2272–86. 27243236 10.1111/1462-2920.13401

[35] Dong C, et al. Berberine, a potential prebiotic to indirectly promote Akkermansia growth through stimulating gut mucin secretion. Biomed Pharmacother. 2021;139:111595. 33862492 10.1016/j.biopha.2021.111595

[36] Guo H, et al. Multi-omics analyses of radiation survivors identify radioprotective microbes and metabolites. Science. 2020. 10.1126/science.aay9097. 33122357 10.1126/science.aay9097 PMC7898465

[37] Popov J, et al. Microbiota-immune interactions in Ulcerative Colitis and Colitis Associated Cancer and emerging microbiota-based therapies. Int J Mol Sci. 2021. 10.3390/ijms222111365. 34768795 10.3390/ijms222111365 PMC8584103

[38] Zhu J, et al. Mechanism of Huanglian Wendan Decoction in ameliorating non-alcoholic fatty liver disease via modulating gut microbiota-mediated metabolic reprogramming and activating the LKB1/AMPK pathway. PLoS One. 2025;20(9):e0331303. 40892742 10.1371/journal.pone.0331303 PMC12404375

[39] Park YH, et al. Adequate dextran sodium sulfate-induced colitis model in mice and effective outcome measurement method. Journal of cancer prevention. 2015;20(4):260. 26734588 10.15430/JCP.2015.20.4.260 PMC4699753

[40] Yao D, et al. MUC2 and related bacterial factors: therapeutic targets for ulcerative colitis. EBioMedicine. 2021;74:103751. 34902790 10.1016/j.ebiom.2021.103751 PMC8671112

[41] Yang Q-Y, et al. Exploring the mechanism of indigo naturalis in the treatment of ulcerative colitis based on TLR4/MyD88/NF-κB signaling pathway and gut microbiota. Front Pharmacol. 2021;12:674416. 34366843 10.3389/fphar.2021.674416 PMC8339204

[42] Calviño-Suárez C, et al. Exploration of JAK/STAT pathway activation in ulcerative colitis reveals sex-dependent activation of JAK2/STAT3 in the inflammatory response. Front Immunol. 2025;16:1609740. 40761789 10.3389/fimmu.2025.1609740 PMC12318950

[43] Babon JJ, Varghese LN, Nicola NA. Inhibition of IL-6 family cytokines by SOCS3. Semin Immunol. 2014;26(1):13–9. 24418198 10.1016/j.smim.2013.12.004 PMC3970923

[44] Zegeye MM, et al. Activation of the JAK/STAT3 and PI3K/AKT pathways are crucial for IL-6 trans-signaling-mediated pro-inflammatory response in human vascular endothelial cells. Cell Commun Signal. 2018;16(1):55. 30185178 10.1186/s12964-018-0268-4 PMC6125866

[45] Long Y, et al. Endoplasmic reticulum stress contributed to inflammatory bowel disease by activating p38 MAPK pathway. Eur J Histochem. 2022;66(2):3415. 35603939 10.4081/ejh.2022.3415 PMC9178311

[46] Baydoun ZA, Rao M, Khan I. Endoplasmic reticular stress and pathogenesis of experimental colitis: mechanism of action of 5-Amino Salicylic Acid. Med Princ Pract. 2025;34(1):39–47. 39496247 10.1159/000541791 PMC11805548

[47] Zhou X, et al. Curcumin improves epithelial barrier integrity of Caco‐2 monolayers by inhibiting endoplasmic reticulum stress and subsequent apoptosis. Gastroenterol Res Pract. 2021;2021(1):5570796. 34659400 10.1155/2021/5570796 PMC8514927

[48] Ghosh S, Pramanik S. Structural diversity, functional aspects and future therapeutic applications of human gut microbiome. Arch Microbiol. 2021;203(9):5281–308. 34405262 10.1007/s00203-021-02516-y PMC8370661

[49] Gasaly N, de Vos P, Hermoso MA. Impact of bacterial metabolites on gut barrier function and host immunity: a focus on bacterial metabolism and its relevance for intestinal inflammation. Front Immunol. 2021;12:658354. 34122415 10.3389/fimmu.2021.658354 PMC8187770

[50] Iyer N, Corr SC. Gut microbial metabolite-mediated regulation of the intestinal barrier in the pathogenesis of Inflammatory Bowel Disease. Nutrients. 2021. 10.3390/nu13124259. 34959809 10.3390/nu13124259 PMC8704337

[51] Ashaolu TJ, Fernández-Tomé S. Gut mucosal and adipose tissues as health targets of the immunomodulatory mechanisms of probiotics. Trends Food Sci Technol. 2021;112:764–79.

[52] Pothuraju R, et al. Mucins, gut microbiota, and postbiotics role in colorectal cancer. Gut Microbes. 2021;13(1):1974795. 34586012 10.1080/19490976.2021.1974795 PMC8489937

[53] Alexander KL, et al. Human microbiota flagellins drive adaptive immune responses in Crohn’s Disease. Gastroenterology. 2021;161(2):522-535.e6. 33844987 10.1053/j.gastro.2021.03.064 PMC8489510

[54] Sorbara MT, et al. Functional and genomic variation between human-derived isolates of Lachnospiraceae reveals inter- and intra-species diversity. Cell Host Microbe. 2020;28(1):134-146.e4. 32492369 10.1016/j.chom.2020.05.005 PMC7351604

[55] Tian B, et al. Lycium barbarum relieves gut microbiota dysbiosis and improves colonic barrier function in mice following antibiotic perturbation. J Funct Foods. 2020;71:103973.

[56] Xie J, et al. Short-chain fatty acids produced by Ruminococcaceae mediate α-Linolenic Acid promote intestinal stem cells proliferation. Mol Nutr Food Res. 2022;66(1):e2100408. 34708542 10.1002/mnfr.202100408

[57] Rao Y, et al. Gut Akkermansia muciniphila ameliorates metabolic dysfunction-associated fatty liver disease by regulating the metabolism of L-aspartate via gut-liver axis. Gut Microbes. 2021;13(1):1–19. 34030573 10.1080/19490976.2021.1927633 PMC8158032

[58] Baldelli V, et al. The role of Enterobacteriaceae in gut microbiota dysbiosis in Inflammatory Bowel Diseases. Microorganisms. 2021. 10.3390/microorganisms9040697. 33801755 10.3390/microorganisms9040697 PMC8066304

[59] Parker BJ, et al. The genus Alistipes: gut bacteria with emerging implications to inflammation, cancer, and mental health. Front Immunol. 2020;11:906. 32582143 10.3389/fimmu.2020.00906 PMC7296073

[60] Ornelas A, et al. Microbial metabolite regulation of epithelial cell-cell interactions and barrier function. Cells. 2022. 10.3390/cells11060944. 35326394 10.3390/cells11060944 PMC8946845

[61] Xu M, et al. Assesment of adulterated traditional Chinese medicines in China: 2003–2017. Front Pharmacol. 2019;10:1446. 31849686 10.3389/fphar.2019.01446 PMC6895211

[62] Wang M, et al. Citrus flavonoids and the intestinal barrier: interactions and effects. Compr Rev Food Sci Food Saf. 2021;20(1):225–51. 33443802 10.1111/1541-4337.12652

[63] Leite CdS, et al. The anti-inflammatory properties of licorice (Glycyrrhiza glabra)-derived compounds in intestinal disorders. Int J Mol Sci. 2022;23(8):4121. 35456938 10.3390/ijms23084121 PMC9025446

[64] Xia L, et al. The effects of phenolic acid supplementation on intestinal barrier function: a review. Food Funct. 2026. 10.1039/D5FO03351A. 41358951 10.1039/d5fo03351a

