# Supplementary material for: Huanglian-Wendan Decoction alleviates DSS-induced colitis by modulating the gut microbiota and protecting against intestinal injury via suppression of colonic apoptosis and endoplasmic reticulum stress
Source: Nat Prod Bioprospect. 2026 Jul 6;16(1):76. doi: 10.1007/s13659-026-00636-w (PMC13338009; doi:10.1007/s13659-026-00636-w)
Supplement: Supplementary file 1 — Supplementary Material 1 [file 13659_2026_636_MOESM1_ESM.docx]

Huanglian-Wendan Decoction alleviates DSS-induced colitis by modulating the gut microbiota and protecting against intestinal injury via suppression of colonic apoptosis and endoplasmic reticulum stress

Liang Li ^a,1^, Xinyi Zhan ^a,1^, Hidayat Ullah ^b, c,d,1^, Wenxian Guo ^a^, Ping Gui ^e,1^, Weijie Peng ^a^, Quanxi Mei ^a^, Weibo Dai ^a,*^, Yuting Duan ^f,*^, Xia Yuan ^g,*^, Xianjing Hu ^b,c,d,e,*^

*^a^ Pharmacology Laboratory, Zhongshan Hospital, Guangzhou University of Chinese Medicine, Zhongshan 528401, Guangdong, PR China*

*^b^ Dongguan Key Laboratory of TCM for Prevention and Treatment of Refractory Digestive Diseases, Guangdong Provincial Key Laboratory of Natural Drugs Research and Development, Guangdong Medical University, Dongguan 523808, PR China*

*^c^ Dongguan Key Laboratory of Fundamental Research and Clinical Application of Toxic Chinese Medicine, The First Dongguan Affiliated Hospital, School of Pharmacy, Guangdong Medical University, Dongguan 523121, PR China*

*^d^ School of Pharmacy, Dongguan Branch, National Engineering Research Center for Modernization of Traditional Chinese Medicine, Guangdong Medical University, Dongguan 523808, PR China*

*^e^ Department of Proctology, The First Dongguan Affiliated Hospital, Guangdong Medical University, Dongguan 523121, PR China.*

*^f^ Evidence-Based Medicine Center, The Affiliated Traditional Chinese Medicine Hospital, Guangzhou Medical University, Guangzhou 510140, PR China*

^g^ Cancer Center, The First Huizhou Affiliated Hospital of Guangdong Medical University, Huizhou 516000, PR China

* *Corresponding authors.*

*E-mail addresses:* huxj2003@163.com (X. Hu), daiweibo007@163.com (W. Dai), duanyt@gzhmu.edu.cn (Y. Duan)

^1^ These authors have contributed equally to this work and share first authorship.

**Supplementary Figure**


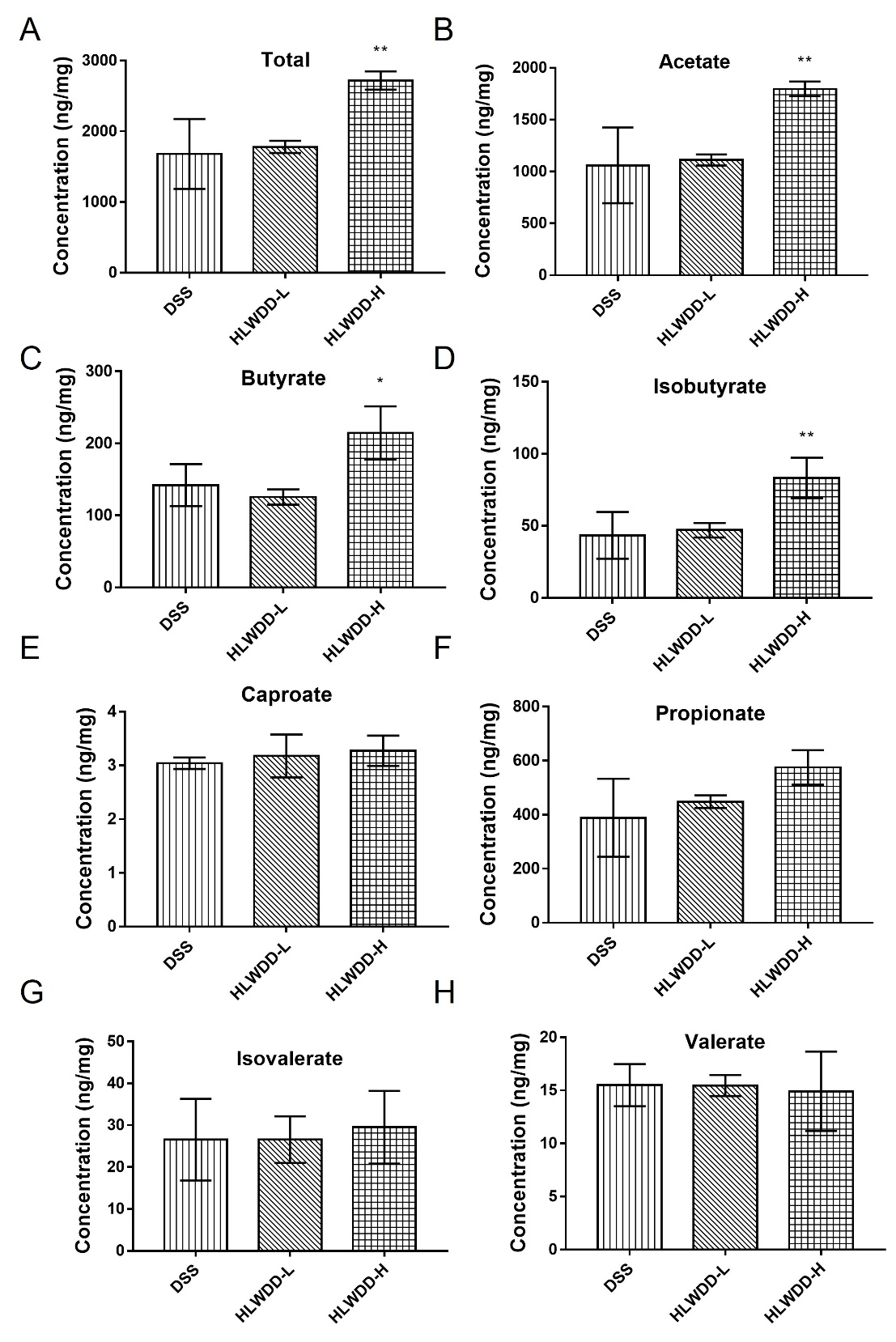


**Figure S1.** Effects of HLWDD treatment on fecal short-chain fatty acid (SCFA) levels and gut microbiota composition. No significant differences were observed in caproate, isovalerate, propionate, or valerate between groups. The increase in SCFAs after HLWDD treatment was primarily limited to acetate, butyrate, and isobutyrate, consistent with the corresponding shifts in the gut microbial community profile.
